# November 11, 2024, T1DX‐QI Learning Session, *Journal of Diabetes* Abstracts

**DOI:** 10.1111/1753-0407.70032

**Published:** 2024-12-10

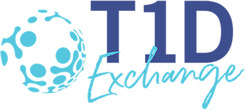

## Age‐appropriate self‐management of type 1 diabetes

### Claire Moore, MD; Naomi R. Fogel, MD; Sean DeLacey, MD

#### Ann and Robert H. Lurie Children's Hospital, Chicago, Illinois, USA

clmoore@luriechildrens.org

**Background:** Euglycemia for pediatric type 1 diabetes (T1D) patients declines following transfer to adult care. Insufficient mastery of diabetes self‐management is a modifiable contributor to the decline. Best‐practice recommendations are lacking on ages to teach diabetes topics and skills. We aim to characterize perspectives of diabetes providers on diabetes self‐management teaching to guide future interventions and improve diabetes self‐management education.

**Methods:** We conducted a written survey of medical professionals involved in T1D care in our pediatric institution. We surveyed their perspectives on ages at which patients should master 22 diabetes topics and 18 skills and their ratings of their comfort and consistency in teaching.

**Results:** Twenty‐three participants (17 physicians, 1 nurse practitioner, and 5 certified diabetes educators) completed surveys. The mean ideal age of transition reported was 19.6 years old. Topics including basic explanation of diabetes, knowledge of hyperglycemia and hypoglycemia symptoms, and glucometer use could be mastered by younger patients (ages 6–10), whereas topics of pregnancy, scheduling appointments, and insurance could be mastered at ages 17–18. Participants reported being comfortable or very comfortable with almost all topics. Most participants indicated that clear guidelines (74%) on ages to address topics and a system (87%) for tracking mastery would increase comfort level in providing education.

**Conclusions:** Data highlight a need for guidance on ages at which topics are taught and methods for tracking patient mastery. Future interventions will incorporate knowledge gained for building guidance and tracking mechanisms for teaching.

**Keywords:** Diabetes mellitus, type 1, self‐management, transition to adult care

## Full‐scale launch of eating disorder screening at a large pediatric diabetes clinic

### Claire Zimmerman, NONE; Rebecca Campbell, BS; Ellen Fay‐Itzkowitz, LCSW, CDCES; Alexander Meyer, BS; Bailey Tanner, BS; Holly K. O'Donnell, PhD; G. Todd Alonso, MD

#### Barbara Davis Center for Diabetes, University of Colorado Anschutz Medical Campus, Aurora, CO, USA

claire.zimmerman@cuanschutz.edu

**Background/Objective**: Routine screening for disordered eating is recommended for adolescents and adults living with type 1 diabetes. We began a clinical pilot in 2023 and used quality improvement methods to scale up disordered eating screening at four pediatric diabetes clinic locations.

**Methods:** The Disordered Eating Problem Survey‐Revised (DEPS‐R) was used to assess disordered eating behaviors in adolescents and young adults ≥12 years. Bi‐weekly multidisciplinary team meetings were convened to improve and expand the process. Training was presented at staff meetings and via email. Additionally, survey results were entered directly into flowsheets with updated electronic medical record (EMR) integration. Upon screening completion, an automated template appeared in providers' notes with screening results and suggested next steps. Positive screening results automatically added referral recommendations to providers' notes.

**Results:** Between October 2023 and August 2024, we administered 1331 disordered eating screenings (Figure). Two hundred fifty (17.8%) scored positive. Initially, screenings were assigned manually with EMR decision support. By June 2024, survey assignment was automated. Providers learned how to discuss screening results with patients and provide resources for behavioral health follow‐up. Automated EMR steps were built to facilitate documentation, appropriate referrals, and billing. Tools were revised periodically to provide patients with up‐to‐date psychological resources.

**Conclusions:** Disordered eating screening is possible with efficient methods that minimize the number of manual steps executed by team members and offer decision support at each step. Automating survey delivery to patients, screen result delivery to the provider during the clinical encounter, and referral and billing facilitation are necessary.

**Keywords:** behavioral health, disordered eating, pediatric, screening, type 1 diabetes

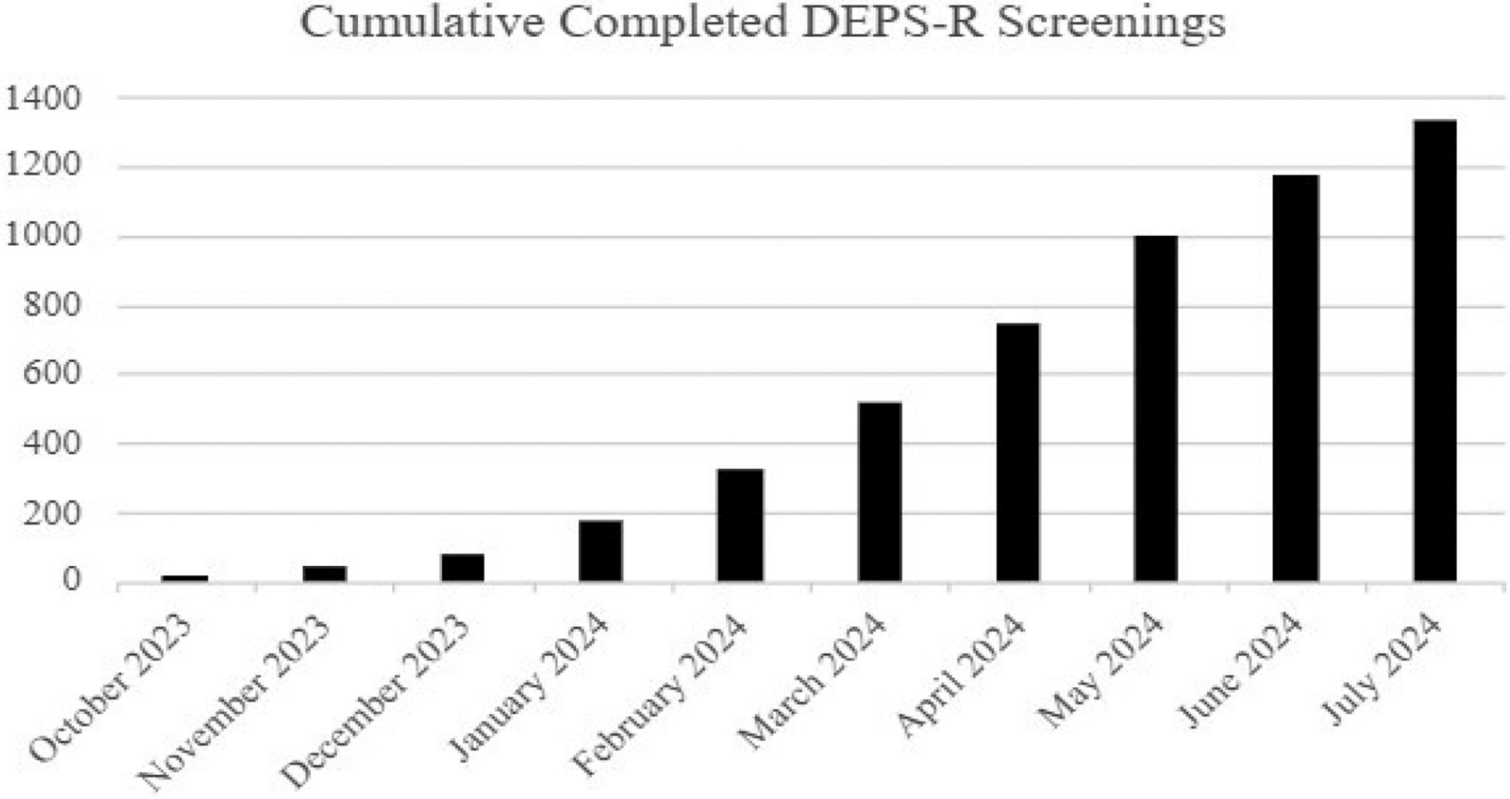

## STRIDE project: Supporting transition readiness in diabetes education

### Sarah Rosenheck, DO, Charlotte Chen, DO, Laurie E. Cohen, MD, Shivani Agarwal, MPH, MD, and Molly O. Regelmann, MD

#### Children's Hospital at Montefiore, Albert Einstein College of Medicine, Bronx, NY, USA

srosenheck@montefiore.org

**Background/Objective:** Transition preparation provides patients with tools for self‐management and improves disease outcomes. Starting education in adolescence addresses knowledge gaps prior to adult care transition. At diagnosis of childhood diabetes mellitus (DM), education is directed to caregivers. The quality improvement (QI) project's objective was to increase discussion and coding for transition readiness by 75% over 12 months at The Children's Hospital at Montefiore Diabetes Clinic.

**Methods:** Data regarding transition discussion were extracted from an electronic medical record (EMR). Multiple Plan‐Do‐Study‐Act cycles were performed to improve transition readiness discussion. After a formal educational presentation about the transition process, eight topics from the Transition Readiness Assessment Questionnaire and Readiness of Emerging Adults with Diabetes Diagnosed in Youth tool were embedded into visit notes. All DM notes were reviewed for a one‐month period. Discussion and ICD‐10 coding rates were then calculated.

**Results:** At baseline, 20% (18/92) of type 1 diabetes (T1D) visits documented discussion of transition readiness, with 5% (46/92) including coding. For type 2 diabetes (T2D), 13% (5/38) of visits documented discussion, with 0% including coding. After including transition readiness questions in the note template, discussion increased to 76% (70/92) for T1D visits and 72% (32/44) for T2D visits, with 45% and 26% coded respectively.

**Conclusions:** Gaps in transition readiness discussion can be reduced by embedding standardized questions into the EMR. Patient education and long‐term DM outcome measures should be assessed in the adult clinic to determine effectiveness of QI interventions during adolescence.

**Keywords:** transition care, diabetes mellitus, quality improvement

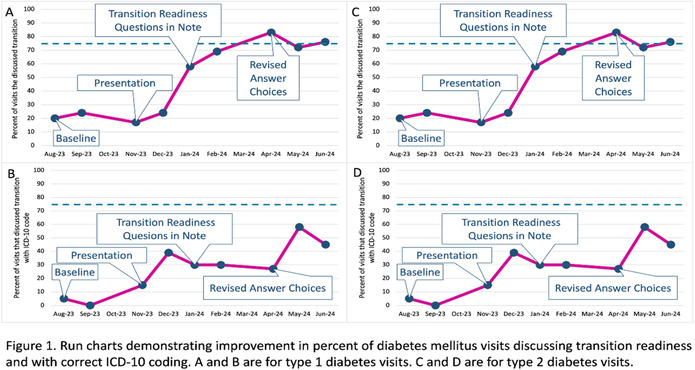

## *Y'ALL* *READDY* *for this?* Embedding transition readiness screening across sites in a pediatric diabetes practice

### Andrew R. Lavik, MD, PhD;^1^ Andrea Mucci, MD, MASc, MEd;^1^ Cheryl Switzer, MSN, CPNP, CDCES;^1^ Alyssa Rowe, MSN, APRN, CPNP‐PC;^1^ Don Buckingham, MBOE, CPHQ, CSSBB^2^

#### ^1^Children's Hospital Institute, Section of Pediatric Endocrinology; Cleveland Clinic, Cleveland, OH, USA; ^2^T1D Exchange, Boston, MA, USA

lavika2@ccf.org

**Background/Objective:** Adolescents with type 1 diabetes (T1D) must eventually transition from pediatric to adult care, a process fraught with challenges. An increasingly used method to evaluate one's preparedness for this transition is the *Readiness Assessment of Emerging Adults with Type 1 Diabetes Diagnosed in Youth (READDY)* tool. To begin refreshing our institution's diabetes transition process to align with the *Six Core Elements of Health Care Transition™*, we designed a quality improvement (QI) project with SMART Aim to increase the percentage of T1D patients aged 15 years and older who receive the READDY tool at visits from 0% to 100% by September 30th, 2024.

**Methods:** With expertise from T1D Exchange QI Collaborative, we employed QI methods to review current practices at our six diabetes clinics. We created a key driver diagram reflecting our theory for improvement. We completed consecutive Plan‐Do‐Study‐Act cycles targeting key drivers and tracked outcomes on a run chart.

**Results:** The percentage of patients 15 years and older with T1D who received the READDY tool increased from 0% to 79% over 9 months. High‐yield strategies included: starting at one site then spreading to all, team role‐specific tip sheets, regular discussion at division meetings, embedding smart phrases in note templates, and standardizing the process for storing completed questionnaires.

**Conclusions:** Using QI methods, we elicited T1D transition readiness in 79% of emerging adults in our practice. We are now studying the 150+ completed surveys to identify themes and targets for future initiatives with plans to monitor progress annually with the READDY.

**Keywords:** quality improvement, transition to adult care, type 1 diabetes

## Design and launch of first pediatric specialty value based program for T1D patients

### Luke Harris^1^; Dominique Pahud^2^; Brent Lockee^3^; Emily L. DeWit^3^ ; Mark Clements^3^

#### ^1^Children's Mercy Integrated Care Solutions, Kansas City, MO USA; ^2^Oros LLC, Kansas City, MO USA; ^3^Children's Mercy Hospital, Kansas City, MO USA

lharris@cmpcn.org

**Background/Objective:** Sustainability of new proactive care models, including Remote Patient Monitoring supported interventions, requires careful planning as Fee‐for‐Service reimbursement often falls short in covering all required activities. To support the ongoing deployment of its proactive care model and associated positive impact on patient outcomes and costs, the Rising T1DE team at Children's Mercy partnered with the region's pediatric Clinical Integrated Network (CIN), operated by Children's Mercy Integrated Care Solutions (ICS), to develop a Specialty Value Based Program for patients with T1D.

**Methods:** A multidisciplinary team was assembled to review potential clinical quality and utilization metrics relevant to a diabetes patient population. The clinical team selected clinical metrics while the CIN team chose utilization metrics. Collaboratively, metric calculation methods, including patient inclusion and exclusion criteria, were established. Performance targets for 2024 were jointly set based on 2019–2023 trends. New dashboards, incorporating both clinical quality and utilization metrics and updated monthly, were built to support active learning and quarterly joint operating meetings.

**Results:** The first Pediatric Specialty Value Based Program for T1D patients was launched in 2024 using performance metrics co‐developed by clinical, operations, data science, and payer/ACO/CIN divisions of the institution and incorporated both infrastructure and performance dependent value‐based funding.

**Conclusions:** Collaborative approaches between clinical, operations, data science, and payer/ACO/CIN teams are essential for the successful design and deployment of Value Based Care Programs and the sustainability of new proactive care models.

**Keywords:** Diabetes Mellitus, Type 1; Pediatric Specialty Value Based Care

**Figure:**
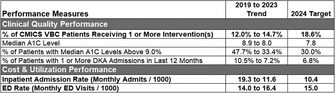
Table 1. Diabetes Value Based Care Program Overview‐ Children's Mercy Integrated Care Solutions (CMICS). Value Based Care (VBC) Program Funding: **Part 1**: Infrastructure Funding; Upfront and Guaranteed. **Part 2**: Clinical Quality and Utilization Metrics Funding: Following evaluation period and Performance based.

## Improving microalbuminuria screening rates among pediatric diabetes patients: A clinic‐wide initiative

### Sarah Lydia Holly, RN, BSN; Jasmine Roberts, BA; Marissa Chotiner, RN, BSN; Shideh Majidi, MD, MSCS

#### Children's National Hospital, 111 Michigan Avenue NW, Washington, DC 20010

sholly@childrensnational.org

**Background/Objective:** Early detection of diabetic nephropathy, through urine screening, is necessary to preventing complications related to chronic kidney disease. Annual screening for microalbuminuria is recommended for all individuals with type 2 diabetes and for those with type 1 diabetes >10 years of age and diagnosed for >5 years. Screening rates at the Diabetes Care Complex at Children's National Hospital have fallen below recommended benchmarks.

**Methods:** An analysis into the clinic's microalbuminuria screening process was conducted and found that the limited capabilities of the electronic medical record and lack of a systematic approach were the main factors contributing to the low screening rate. The team implemented a Plan‐Do‐Study‐Act approach with two successive cycles. In the first, an external dashboard was shared with providers to identify patients due for screening. In the second cycle, the dashboard was shared with the head clinic nurse who then integrated screening into the patient triage process. A statistical control chart tracked monthly screening rates.

**Results:** After 12 months, microalbuminuria screening rates increased from a baseline average of 49% to 76% (Figure 1).

**Conclusions:** This initiative demonstrates that a series of simple interventions can significantly impact patient care and quality outcomes. The systematic identification of patients due for screening and integration of the screening process into routine clinical workflows were pivotal to increasing rates. These findings suggest that similar approaches can be applied to other lab screening efforts to improve outcomes, efficiency, and patient satisfaction.

**Keywords:** Pediatric diabetes, quality improvement, screening

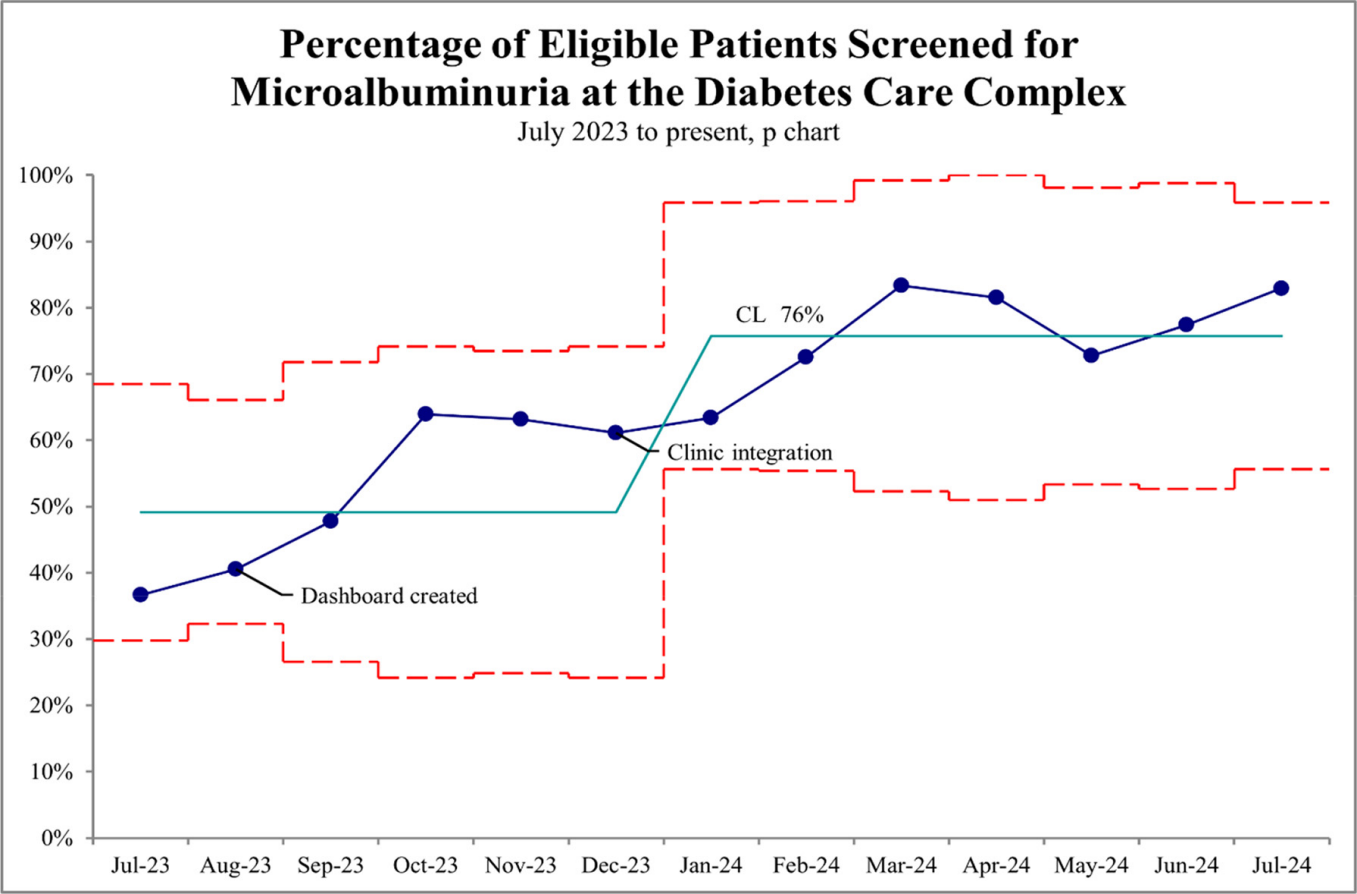

## Addressing disparities in diabetes care: Implementing SDOH screening at diagnosis

### Sarah Lydia Holly, RN, BSN; Jasmine Roberts, BA; Lauren DeAnna, LICSW; Roxanna Velasquez, BA; Shideh Majidi, MD, MSCS

#### Children's National Hospital, 111 Michigan Avenue NW, Washington, DC 20010

sholly@childrensnational.org

**Background/Objective:** Social determinants of health (SDOH) significantly influence diabetes management and outcomes. Despite advancements in care, disparities persist, particularly among marginalized populations. This project aims to implement SDOH screening at the time of diagnosis in order to identify and address these disparities early.

**Methods:** Children's National Hospital launched a standardized SDOH screening at the time of diabetes diagnosis in September 2023. The screen targets 10 domains of SDOH and is administered by Social Work. Caregivers who identify a barrier(s) are provided with a list of tailored resources and ongoing support from the Diabetes Health Coach. Comparisons among new onset diabetes patients (NODPs) were made to assess demographic patterns and prevalence of identified barriers.

**Results:** 162 NODPs were seen between September 2023 and July 2024 ( **Race/Ethnicity** : 41% Black, 31% Other, 27% White; 77% Non‐Hispanic, 16% Hispanic, 7% Unknown; **Insurance** : 51% Commercially insured, 42% Medicaid, 7% Uninsured; **Diagnosis** :78% T1D, 22% T2D). 76% (*n* = 125) of eligible NODPs were screened. 34% (*n* = 87) of participants identified at least one SDOH barrier ( **Race/Ethnicity** : 50% Black, 37% Other, 13% White; 63% Non‐Hispanic, 27% Hispanic; **Insurance** : 30% Commercial insurance, 57% Medicaid, 13% Uninsured; **Diagnosis** :77% T1D, 23% T2D). All participants who identified a SDOH barrier(s) received individualized resources. Mental health and food insecurity were among the most identified barriers.

**Conclusions:** The implementation of SDOH screening demonstrates a proactive approach to addressing barriers and further reiterates the prevalence of disparities. By identifying and responding to these challenges early, we are working to promote more equitable care.

**Keywords**: equity, pediatric diabetes, social determinants of health, quality improvement

## Assessing readiness to transition to adult care among young adults with T1D

### Jody Beth Grundman, MD, MPH; Amanda Perkins, CPNP, CDCES, MPH; Sarah Lydia Holly, BSN, RN; Mai Tran, PharmD, BCACP, BCGP, CDCES; Rachel Longendyke, MD; Julie Harlam, CPNP; Alyssa Danner, BSN, RN; Jennifer Reilly, RD, CDCES; Shideh Majidi, MD, MSCS

#### Children's National Hospital, Washington, DC, USA

jgrundman@childrensnational.org

**Background:** The transition from pediatric to adult healthcare for those with Type 1 Diabetes (T1D) can be challenging. Assessing transition readiness is crucial to support this process. We modified the READDY questionnaire to enhance its usability among young adults with T1D.

**Methods:** A 29‐item modified READDY questionnaire was pilot‐tested by a staff member with diabetes of similar age to the target population, confirming its clarity and feasibility with an average completion time of 2.5 minutes. Our initial Plan‐Do‐Study‐Act (PDSA) cycle involves administering the survey to 18–20‐year‐olds with T1D in a single outpatient clinic setting through two providers. We scored each section and tracked baseline scores and survey distribution rates.

**Results:** Of the 95 young adults scheduled for clinic visits over the next 6 months, 6 met eligibility for the first PDSA cycle. 83.3% of eligible respondents (*n* = 5/6) completed the questionnaire in the first 2‐week PDSA cycle. Median scores among respondents were: knowledge 75%, navigation 90.3%, insulin management 91.7%, health behaviors 80%, and pump skills 100%. Preliminary results will assess distribution feasibility and initial scores.

**Conclusions:** This quality improvement initiative seeks to enhance the transition experience for young adults with T1D by refining the READDY questionnaire and systematically identifying and addressing key barriers to transition readiness. Future steps include expanding distribution by involving more providers, extending to satellite clinics, and broadening the age range of participants. Baseline score evaluation will guide addressing barriers and prioritizing intervention development.

**Keywords:** diabetes mellitus, type 1, quality improvement, transition to adult care, young adult

## Developing a tracking tool for insulin pump prescriptions among children and adolescents with type 1 & type 2 diabetes

### Amanda Perkins, CPNP, CDCES, MPH; Mai Tran, PharmD, BCACP, BCGP, CDCES; Jody Grundman, MD, MPH; Sarah Lydia Holly, RN, BSN; Hadley Kessenich, RD, CDCES; Shideh Majidi, MD, MSCS

#### Children's National Hospital, Division of Endocrinology, 111 Michigan Ave NW, Washington, DC, 20010

aperkins@childrensnational.org

**Background/Objective:** Disparities exist in rates of insulin pump uptake despite evidence that use improves glycemic outcomes. Successful pump uptake requires a multi‐disciplinary team of prescribers, pharmacists, diabetes educators and administrative staff. The ability to track the process can increase uptake.

**Methods:** Inability to track pump initiation through education, prescription fulfillment and pump initiation was identified in a key driver diagram as a barrier to uptake. Process mapping was completed with input from a multi‐disciplinary team. Iterative Plan‐Do‐Study‐Act cycles were undertaken to develop a Pump Powerform for use as a tracking tool. The Powerform was integrated into the Cerner electronic medical record (EMR), trialed with key staff for 2 months, expanded to two Powerforms based on user feedback and rolled out department wide.

**Results:** The Pump Request Powerform (PRPF) and Pump Education Powerform (PEPF) were developed to be accessible for documentation by a multi‐disciplinary team. The PRPF is documented by pharmacy and refill teams and has separate tabs which allow for documentation of insurance‐required record submission, prior authorizations and appeals on medical and pharmacy benefits. The PEPF allows the pump prescriber to indicate patient‐specific characteristics pertinent to pump education (language, social situation, history of tech use and competency with diabetes self‐management skills). A total of 132 Powerforms were initiated since inception (Figure).

**Conclusions:** Pump Powerforms embedded in the EMR create a centralized location to document new pump prescriptions, allow a multi‐disciplinary team to follow progress, and prepare the diabetes educator to deliver customized education, optimizing successful patient transition to technology.

**Keywords:** insulin pumps, quality improvement, technology, pediatric diabetes

**Figure:**
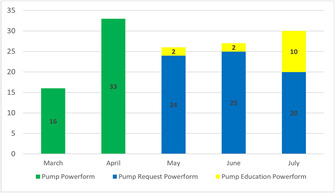
Figure: Pump Powerform Usage March–July 2024

## ConnecT1D data visualization: Informing interventions and equitable improvement in outcomes for patients with type 1 diabetes (T1D)

### Amanda Howell, MPH, CPH; Nana‐Hawa Yayah Jones, MD; Patrick W. Brady, MD, MSc; Michelle I. Knopp, MD; Amy Grant, DNP, RN, CPN; Laura Smith, PhD, CDCES; Amanda Riley, MS, RD, LD, CDCES, Marissa Town, BSN, RN, CDCES; Sarah Corathers, MD

#### Cincinnati Children's Hospital Medical Center, Cincinnati, Ohio, USA

amanda.howell@cchmc.org

**Background/Objective:** ConnecT1D is a quality improvement (QI) initiative focused on enhancing and prioritizing equitable T1D outcomes. Effective data visualization of near‐real time data is crucial for identifying successful interventions and monitoring equity performance.

**Methods:** We identified seven priority processes and outcome measures to access on demand. We developed a self‐service dashboard using Microsoft PowerBI, selected for its ability to integrate and transform data from multiple sources, including our electronic health record. It also allows data to be merged, transformed, linked for calculation, and utilized at patient‐level. The EasySPC app was employed for statistical process control time series charts, allowing analysis across the center population and various subgroups (e.g., race and insurance).

**Results:** Our HIPAA compliant dashboard is now operational, accessible via sharable links, subscription, and Microsoft Teams. It features 118 visualizations, 15 measures, and over 20,000 data points spanning 2488 patients and 3.5 years. Measures include healthcare access, psychosocial support, technology uptake, HbA1c, and Time in Range. Each outcome can be stratified by age, insurance type, race/ethnicity, and diabetes duration. Notably, data visualization highlights that adoption of automated insulin delivery systems (AIDs) is associated with over 1% reduction in mean HbA1c for both public and privately insured youth, narrowing health equity gaps.

**Conclusions:** Effective data visualization clearly demonstrates compelling changes at a population level and across important subgroups. Care centers and care teams can maintain a feedback loop to continuously refine insights and interventions to improve patient outcomes.

**Keywords:** dashboards; data visualization; diabetes mellitus; health equity; type 1

**Figure:**
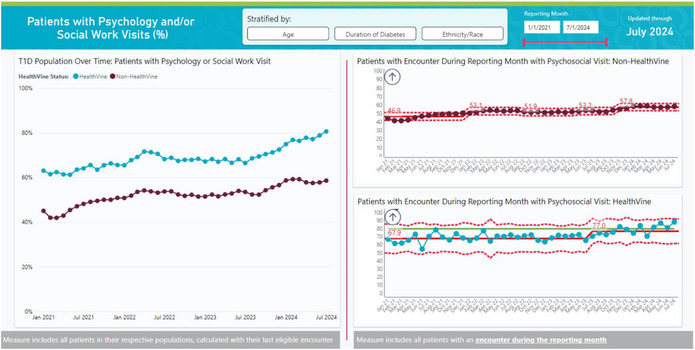
**Figure 1**. Interactive data visualization of rates of AID uptake over time stratified by insurance type. Rates of AID uptake for both Healthvine Ohio Medicaid in blue and privately insured youth with T1D in maroon increased over time and are associated with HbA1c lowering compared to non‐AID use.

## ConnecT1D: Proactive outreach intervention to improve equitable care for youth with type 1 diabetes

### Jennifer J. Kelly, APRN; Yoori Noh, APRN; Siobhan Tellez, DNP, APRN; Amy Grant, DNP, RN, CPN; Amanda Howell, MPH, CPH; Gajanthan Muthuvel, MD; Patrick W. Brady, MD, MSc; Sarah Corathers MD

#### Cincinnati Children's Hospital Medical Center, Cincinnati, Ohio, United States

Jennifer.Kelly@cchmc.org

**Background/Objective:** A single center APRN‐led pilot program of proactive outreach involving a cohort of high‐risk youth (HbA1c >10%) was trialed as part of a larger equity‐based project to improve health outcomes for youth with type 1 diabetes (T1D).

**Methods:** A monthly outreach list identified Medicaid‐managed youth with HbA1Cs >10%. Each month we focused on approaching youth whose birthday fell within that month. Initial contact was performed in person, if possible, followed by secure messaging, phone, and text. The APRN initiated proactive outreach using the method chosen by the family, and without pre‐determined objectives. Families who chose to join the proactive outreach program were contacted at 2‐week intervals; more frequently if identified by the family and/or APRN. The contact interval lengthened if there was sustained improvement. A shared, color‐coded calendar tracked all outreach.

**Results:** 24 individuals were offered proactive outreach from January to July 2024, and 22 (92%) agreed to participate. One person withdrew after enrolling. All individuals who were enrolled experienced improvement in HgbA1c or GMI within 2 months with a mean of 2.7% (median 2.5%). Support given during contact included insulin adjustments, prescription assistance, device support, and general diabetes support/teaching topics.

**Conclusion:** Proactive personal outreach to families resulted in substantially improved glycemic outcomes. The outreach program was acceptable to families and feasible to conduct as an adjunct to routine care. Results from this pilot will be tracked to confirm sustained improvement and design a scalable intervention for a larger population of youth with T1D.

**Keywords:** advanced practice nursing, adolescent, insulin, type 1 diabetes

**Figure:**
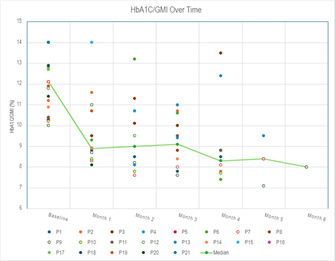
Figure: A median decrease in HbA1c or GMI of 2.5% observed within 2 months of enrollment in the pilot proactive reach out cohort.

## Moving on up: Employing a mobile care center to enhance access to care for youth with type 1 diabetes

### Gajanthan Muthuvel, MD; Bliss Magella, PhD; Amanda Howell MPH, CPH; Amanda Riley, MS, RD, CDCES; Marissa Town, BSN, CDCES; Rebecca Taylor, BS; Nana‐Hawa Yayah Jones, MD; Patrick W. Brady, MD, MSc; Laura Smith, PhD, CDCES; Sarah Corathers, MD

#### Cincinnati Children's Hospital Medical Center, Cincinnati, OH, USA

gajanthan.muthuvel@cchmc.org

**Background/Objective:** Distance from clinics poses a significant barrier to care for youth with type 1 diabetes (T1D), particularly in rural areas. As part of a multi‐faceted quality improvement project, ConnecT1D, we aimed to employ a mobile care center (MCC) to bring in‐person care closer to homes for T1D youth at‐risk.

**Methods:** We equipped an existing Cincinnati Children's MCC to provide standard diabetes care. Geocoding was used to map patients' residential zip codes and overlay rates of missed clinic visits and diabetes‐related hospital admissions to identify need. A location in a neighboring county with an existing school nurse partnership was selected for a monthly outreach clinic. Outcomes measured included completed visits, use of diabetes technology, patient/family experience, and hemoglobin A1c (HbA1c).

**Results:** Across 13 clinics, 25 unique patients with T1D were seen for 49 provider and 35 diabetes educator visits. Most patients resided within the same zip code as the MCC location. One patient initiated CGM and four started automated insulin delivery (AID) systems. Among 9 individuals with repeat visits, mean HbA1c decreased from 9.5% to 9.1%. Notably, a previously lost to follow‐up patient experienced a dramatic HbA1c reduction from >13.5% to 6.5% over two MCC visits after initiating AID.

**Conclusions:** Mobile care clinics can deliver meaningful diabetes care closer to home and improve glycemic outcomes. Future work will target increasing capacity of the MCC and tracking ongoing glycemic improvements.

**Keywords:** access to care, type 1 diabetes, mobile health

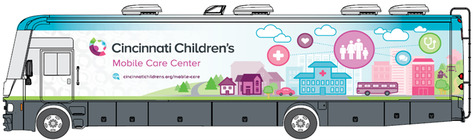

## Increasing screening for social drivers of health in pediatric diabetes

### Barbara Liepman, RN MS CDCES CHWC; Angel Nip, MD; Jenise C. Wong, MD PhD

#### Department of Quality and Patient Safety and Department of Pediatrics, Benioff Children's Hospitals, University of California San Francisco, San Francisco, CA USA

barbara.liepman@ucsf.edu

**Background/Objective:** Social drivers of health (SDoH) include factors such as food and transportation insecurity. Awareness of SDoH can help diabetes care teams address the impact of these factors on patients' health. The objective of this project was to increase screening for SDoH (food insecurity, transportation) in patients seen in the two main diabetes clinics at Benioff Children's Hospitals from a baseline of 11% (fiscal year FY23 4th quarter average) to >50% by the end of FY24 (4th quarter average).

**Methods:** A survey of diabetes team members was conducted to assess knowledge and perceived importance of SDoH screening. A multidisciplinary Task Force (TF) was established in April 2023 to identify, test, and implement change ideas. The TF prioritized using existing screening tools in the electronic medical record (EMR) and standardizing screening workflows for in‐person visits. A list of food and transportation resources was compiled for patients with positive screens. Patients could speak with a team member if they needed additional SDoH resources.

**Results:** Screening for SDoH started in July 2023 with five physicians and nurse practitioners in English. It expanded to include all languages and patients in September 2023. An EMR‐based platform was introduced in April 2024 to refer families to community resources. The average screening rate for the 4th quarter of FY24 was 66% (Figure).

**Conclusions:** Systematic screening for SDoH at in‐person pediatric diabetes visits was successfully implemented, and resources were provided to families in need. Next steps include expanding screening to video visits and/or via patient portals.

**Keywords:** child; diabetes mellitus, type 1, type 2; food insecurity; health inequities; quality improvement

**Figure:**
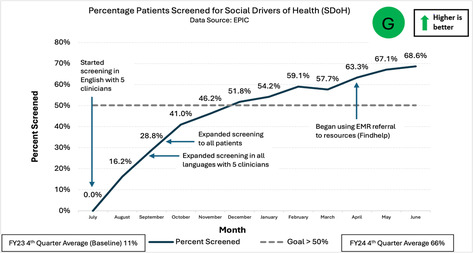
**Figure:** Run chart of percentage of patients in two pediatric diabetes clinics screened for food and transportation insecurity.

## Standardizing insulin pump back‐up plans: Improving documentation and patient confidence through quality improvement

### Kai E. Jones, Sister Grace Mirian Usala, Alyssa Carvalho, Doriann Klaassen, Cynthia J. Herrick, Natalia Genere

#### Division of Endocrinology, Metabolism, & Lipid Research, Washington University School of Medicine, St. Louis, MO, USA

kaijones@wustl.edu

**Objective:** Insulin pump therapy has revolutionized T1D management but increases the risk of diabetic ketoacidosis. To mitigate ketoacidosis, professional societies recommend insulin pump failure plans. We instituted a quality improvement project to (1) standardize documentation and (2) evaluate patients' confidence in their personalized back‐up plan.

**Methods:** A retrospective review was conducted to determine frequency of patients having the necessary components of pump back‐up plan (abbreviated as “WIS”), including (1) **W**ritten back‐up plan with dosing, (2) intermediate or long‐acting **I**nsulin prescription, and (3) appropriate injection **S**upplies. Clinician and patient surveys gathered insights into back‐up plan practices. A single page ‘Back‐up Plan’ document was developed with troubleshooting and patient‐specific dosing of insulin. We then assessed several interventions for increasing utilization of the ‘Back‐Up Plan’.

**Results:** In a baseline assessment of 89 patients, 47% had no documented insulin pump back‐up plan and only 33% had all WIS components present. CDCES visits were associated with higher likelihood of successful WIS components (53.3% vs. 11.4%, *p* < 0.001). Yet only 39% of patients had a CDCES visit within the year. Barriers to scheduling CDCES visits led to the creation of a multidisciplinary technology clinic. After 6 months (*n* = 13), 96% of patients had a CDCES visit, and 54% had all WIS components (*n* = 26). Patient confidence in their back‐up plans increased from 57% (*n* = 17) as compared to 85 post‐interventions (n = 13).

**Conclusions:** Standardizing back‐up plan documentation and increasing CDCES engagement through a multidisciplinary clinic improved WIS component availability and boosted patient confidence in their back‐up plans.

**Keywords**: diabetes mellitus, type 1/complications, insulin infusion systems/ adverse effects, quality improvement

## Optimizing automated insulin delivery system use in youth with recent onset T1D

### Mili Vakharia, FNP‐C, CDCES, Daniel J. DeSalvo, MD, Sarah K. Lyons, MD, Don Buckingham, MBOE, CPHQ, Sarah Kelly, DNP, NP‐C, Siripoom McKay, MD, Rona Sonabend, MD, Grace Kim, MD

#### Division of Pediatric Diabetes and Endocrinology, Pediatrics, Baylor College of Medicine/Texas Children's Hospital, Houston, Texas, USA

vakharia@bcm.edu

**Background:** Automated insulin delivery (AID) systems improve glycemic outcomes and burden of care in individuals with type 1 diabetes (T1D). Guidelines recommend AID for youth with TID with standardized technology education to optimize success with device use. We implemented a quality improvement initiative aimed at increasing AID system use in all recent onset T1D patients, less than 1 year from diagnosis, by 20% from baseline of 1.2%, by July 2024.

**Methods:** A series of Plan‐Do‐Study‐Act cycles were implemented including:

1. Implementing a new process for early introduction of insulin pumps within 90 days of T1D diagnosis, including all patients receiving an “insulin pump action plan” (June 2022).
2. Scheduling a 2‐week post‐diagnosis telemedicine visit with a provider and diabetes educator (October 2022).
3. Hosting a department level “pump workshop” educating diabetes educators and providers, including reminders about documentation (April 2023).
4. Standardizing AID system starts across our 6 diabetes clinics, including scheduling initial post‐pump visits within 30 days of pump start, and weekly phone contact with diabetes educator for those new to pump (October 2023).
5. Sending bulk patient portal message to families about AID systems covered by Texas Medicaid (July 2024).

**Results:** In patients with T1D duration of Al year, AID system usage has increased from a baseline of 1.2% in June 2022 to over 30% in July 2024 and remains sustained (Figure 1).

**Conclusion:** Structured and multitiered robust support is essential for successful onboarding of new diabetes technology.

**Keywords**: AID, insulin pumps, T1D, QI

**Figure:**
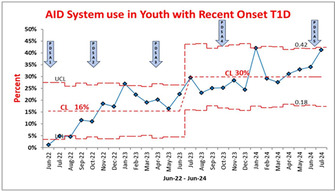
Figure 1. AID system use in youth with recent onstet T1D.

## Use of non‐insulin medication in youth with T2D

### Mili Vakharia, FNP‐C, CDCES, Maria Diaz, RND, LD, CDCES, Siripoom McKay, MD, Don Buckingham, MBOE, CPHQ, Sarah Lyons, MD, Rona Sonabend, MD, Grace Kim, MD

#### Division of Pediatric Diabetes and Endocrinology, Department of Pediatrics, Baylor College of Medicine/Texas Children's Hospital, Houston, Texas, USA

vakharia@bcm.edu

**Background:** Youth onset of type 2 diabetes (T2D) required tailored medication management that is different from type 1 diabetes. American Diabetes Association guidelines recommend use of newer therapies such as glucagon‐like peptide 1 receptor (GLP‐1) and/or sodium glucose cotransporter‐2 (SGLT‐2) inhibitors in youth with unmet glycemic control. Our quality improvement initiative aimed at increasing the percentage of non‐insulin medications for youth with new onset T2D (<1 year of diagnosis) by 5% from baseline of 74% by July 2024.

**Methods:** The following Plan‐Do‐Study‐Act cycles were implemented:

1. Developed a multidisciplinary FRAME Works T2D education program (**F**ollow a balanced meal, **R**educe insulin resistance with lifestyle change, **A**dhere to medication, **M**onitor blood glucose & comorbidities, and **E**ngage support system) (Aug 2022).
2. Educated providers and ancillary staff on new FRAE program (May 2023).
3. Implemented the FRAME program for inpatient new onset T2D education, including discharge knowledge assessment (SEP 2023).
4. Adopted FRAME program for outpatient new onset T2D education (January 2024).
5. Created T2D medication guide for providers (February 2024).
6. Hosted a division workshop on T2D management (June 2024).

**Results:** P‐chart showed shift in center line with percentage of patients with T2D, <1 year of diagnosis, on a non‐insulin medication increased from baseline of 74% to 83%, from June 2022 to July 2024 (Figure 1).

**Conclusions:** Our project facilitated interprofessional‐lead patient education for youth with T2D using FRAME and improved rates of non‐insulin medications. In the future, we aim to evaluate changes in HbA1c rates.

**Keywords**: type 2 diabetes, youth, quality improvement

**Figure:**
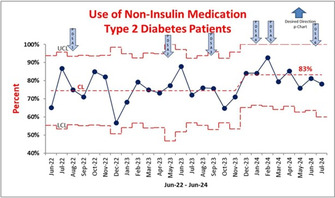
Figure 1. Youth with T2D on non‐insulin medication

## Reducing disparities in continuous glucose monitor adoption and use among children and adolescents with type 1 diabetes

### Ashley Garrity, MPH; Jacqueline Fisher, MD; Inas Thomas, MD; and Joyce Lee, MD, MPH

#### Division of Pediatric Endocrinology & Susan B. Meister Child Health Evaluation and Research (CHEAR) Center, Department of Pediatrics, Michigan Medicine, University of Michigan, Ann Arbor, Michigan, USA

ashleyna@med.umich.edu

**Background:** Despite benefits of continuous glucose monitors (CGMs) for individuals with type 1 diabetes (T1D) disparities in adoption and use by race/ethnicity persist. In February 2022, our clinic noted lower CGM adoption among non‐White T1D patients (75%) compared to non‐Hispanic White (NHW) T1D patients (87%). We aimed to increase CGM adoption and use and reduce disparities between NHW and non‐White patients.

**Methods:** Using the T1D Exchange Equity Framework, we identified root causes and process pain points and implemented plan‐do‐study‐act (PDSA) cycles:

1. Provided patient instructions for obtaining CGM and contacting medical supply companies.
2. Offered in‐clinic support for CGM initiation/placement.
3. Revised electronic health record (EHR) flowsheet to better track CGM adoption, usage, and barriers.
4. Downloaded additional data for patients using only glucometers, ensuring sufficient order documentation.
5. Added CGM resources to patient/family e‐newsletter.
6. Advertised CGM in new‐onset education class.
7. Assisted newly diagnosed inpatients with CGM app setup and data sharing.

Additionally, we engaged in Medicaid CGM coverage advocacy, resulting in improved accessibility.

**Results:** By June 2024, 98% of NHW and 97% of non‐White T1D patients had CGMs (Figure 1A). When we began collecting data on CGM use in February 2023, 78% of NHW and 84% of non‐White T1D patients with a CGM used it at least 10 of the last 14 days; By June 2024, usage increased to 89% and 88%, respectively (Figure 1B).

**Conclusions:** Increasing CGM adoption and use while also reducing racial/ethnic disparities requires a multi‐pronged and tailored approach to address individualized barriers.

**Keywords:** continuous glucose monitoring; diabetes mellitus, type 1; health equity

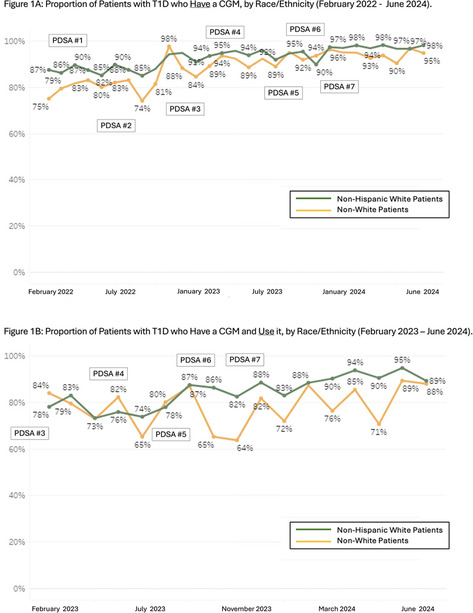

## Improving continuous glucose monitors prescribing behaviors in primary care

### Jovan Milosavljevic, MD^1^ ; Rohan Maini, MD^2^ ; Jing‐Yu‐Pan, MD^2^ ; Priyanka Mathias, MD^1^ ; Justin Mathew, MD^1^ ; Michael Greenberg, NP^1^ ; Sarah Baron, MD, MS^2^ ; Sharon Rikin, MD, MS^2^ ; Shivani Agarwal, MD, MPH^1^

#### ^1^Fleischer Institute of Diabetes and Metabolism, Division of Endocrinology, Montefiore Medical Center, Albert Einstein College of Medicine, Bronx, New York, USA. ^2^Department of Medicine, Montefiore Medical Center, Albert Einstein College of Medicine, Bronx, New York, USA

jmilosavlj@montefiore.org

**Background/Objective:** Despite being the standard of care for people with diabetes on insulin, continuous glucose monitoring (CGM) remains underutilized in primary care settings, yet it has great potential to improve population level glycemic and quality of life outcomes. We aimed to increase CGM prescription rates for adults with insulin‐treated diabetes at Montefiore Internal Medicine clinic by 10% from 9/2023 to 6/2024.

**Methods:** Discovery work included assessing baseline prescription rates and identifying targets for intervention. Based on prior QI initiatives in Montefiore Endocrinology, we spread learnings to primary care through: (1) designing standardized prescription process detailing eligibility criteria, pharmacy selection, and prior authorization; (2) organizing tailored education sessions for faculty on CGM technology, eligibility, data access, and interpretation; (3) organizing trainings of clinic champions and diabetes educators (CDE) by technology company representatives; and (4) reserving slots for CDEs and clinicians to support patients in CGM placement and education. We calculated CGM prescription rates as the number of people with a CGM prescription in the reporting month, divided by total number of insulin‐treated patients with an office visit in the same month.

**Results:** After PDSA cycles, CGM prescription rates increased from a median baseline of 6.7% to a new median of 11.4% (relative increase of 70%).

**Conclusion:** Optimization of prescription processes and targeted education in primary care can increase CGM prescribing rates. While these interventions led to a small increase in prescription rates over a short period, further scaling and sustained efforts are necessary to achieve more significant and widespread impact.

**Keywords:** continuous glucose monitoring; primary care; diabetes technology

## Ongoing efforts for increasing retinopathy screening at a pediatric diabetes center

### Jeniece Ilkowitz RN, MA, CDCES, Rebecca Chong, RN, Mary Pat Gallagher, MD

#### Hassenfeld Children's Hospital at NYU, Pediatric Diabetes Center New York, NY, USA.

jeniece.ilkowitz@nyulangone.org

**Background/Objective:** In 2024, a Quality Improvement (QI) project began to increase rates of retinopathy screening at a NYC Pediatric Diabetes Center (PDC). In this QI, we aimed to increase documented retinopathy screening of eligible patients living with type 1 diabetes (T1D) by 10% in 6 months after acquiring an Optos ultra‐widefield Retinal Imagine Device (Optos).

**Methods:** The Optos is utilized to provide retinopathy screening for eligible patients; T1D ≥5 years and no retinopathy screen in the past 1–2 years, self‐reported by patient or documented in EMR. Multiple Plan‐Do‐Study‐Act (PDSA) cycles were performed to optimize use of the Optos. PDSA cycles included: staff training, education materials, creating EMR schedule for same day screenings, youth and family education, EMR note templates, and streamlined reading of the screen by ophthalmologist covered by insurance.

**Results:** Baseline monthly percentage of patient visits at the PDC with documented retinopathy screening in the past 2 years was on average 41.3%. Following Optos set‐up and multiple PDSA cycles, documented monthly percentage of patient visits with retinopathy screening in the past 2 years increased to 52% over 6 months.

**Conclusions:** The Optos, along with multiple initiatives, helped increase documentation of and on average increased retinopathy screening rates. Clinicians reported improvement in workflow, timeliness of results and patient satisfaction. Future efforts should identify barriers to youth getting retinopathy screening at the PDC, and QI initiatives to continue increasing screening rates and documentation by all PDC providers.

**Keywords:** diabetes mellitus, diabetes mellitus, type 1, retinopathy, quality improvement

## Family centered team meetings for people with HbA1c > 9% for > 12 months

### Jeniece Ilkowitz, RN, MA, CDCES, Vanessa Wissing, RD, CDCES, Mary Ann Harris, LCSW, Mary Pat Gallagher, MD

#### The Hassenfeld Children's Hospital, Pediatric Diabetes Center at NYU Langone, New York, NY, USA

jeniece.ilkowitz@nyulangone.org

**Background/Objective:** The HCH Pediatric Diabetes Center (PDC) created a Wellness Program to provide supports to people with HbA1c >9%. In a previous Quality Improvement (QI) project, we noted success for 39% of those enrolled. The aim of the current QI is to decrease HbA1c of children with chronically elevated (CE) HbA1c, who were identified as appropriate candidates for a Family Centered Team Meeting (FTCM), by 20% over 6 months.

**Methods:** This QI began January 2024. First, a workgroup reviewed each CE case (*n* = 33), defined as an HbA1c >9% for >12 months. Of these, nine (27%) were identified to participate in a FCTM due to the involvement of multiple caretakers and/or need of resources in the community. The goal of the FCTM was to elicit perspectives of all involved, (child, family, PDC, community partners), and develop a comprehensive, mutually agreed upon plan.

**Results:** Of the nine, four (44%) participated in a FCTM (of the remaining five, there were two sets of siblings). HbA1c decreased on average by 11.3% (SD −33.4) over 6 months for FCTM children versus 4% (SD −13.6.7.7) of those who did not attend a FCTM.

**Conclusions:** This QI effort decreased average percent HbA1c by 11.3% in a cohort of children with CE HbA1c who attended a FCTM. Ongoing initiatives include further investigation into how to engage additional families and accomplish goals. Barriers identified for this group included: additional medical diagnoses, housing insecurity, and barriers obtaining diabetes technology supplies. Ongoing efforts will further review barriers and interventions for the entire CE population.

## Implementation of the ASQ suicide risk screening in routine pediatric diabetes care

### ^1^Risa M Wolf, MD, ^1^Saleel Fatima, MD, ^2^Laura Prichett, PhD, ^1^ ^3^Nancy Campbell, MSW, LCSW‐C, ^4^Meg C.N. Snyder, PsyD, ^4^Morgan Bifano, PsyD ^4^, ^1^ Elizabeth Brown, MPH

#### Division of Pediatric Endocrinology, Johns Hopkins University School of Medicine

Baltimore, MD, USA

rwolf@jhu.edu

**Background:** Depression and suicide are more prevalent in adolescents with chronic illnesses such as diabetes. Psychosocial assessment is recommended in routine diabetes care. This QI project's goal was to determine the prevalence of suicide risk in youth with diabetes through the Ask Suicide‐Screening Questions (ASQ) and the Patient Health Questionnaire‐9 Item‐9 (PHQ‐9).

**Methods:** The PHQ‐9 and ASQ were prospectively administered to patients with Type 1 Diabetes (T1D) and Type 2 Diabetes (T2D), ages 11–24 years at routine diabetes visits at a pediatric diabetes center from January to December 2023. Depression and suicide risk were assessed using PHQ‐9(item 9) and ASQ. The sensitivity and specificity of PHQ‐9 was determined using ASQ as the reference standard.

**Results:** Among the 309 patients screened, 237(76.6%) had T1D and 72(23.3%) had T2D. The mean age was 15.1 ± 2.6 years, 145 (46.9%) were female, and mean HbA1c was 8.6 ± 2.3%. The prevalence of suicide risk using PHQ‐9 item 9 was 5.9% in T1D and 12.5% in T2D, and 8.4% in T1D and 19.4% in T2D, using ASQ. After a positive suicide risk screen, only 52.9% completed recommended mental health follow‐up within 1 month.

**Conclusions:** Prevalence of suicide risk is higher in youth with Type 2 diabetes compared to Type 1 Diabetes. PHQ‐9 is less sensitive in identifying suicide risk in adolescents and young adults compared to the ASQ. Diabetes care teams should consider using specific suicide risk screeners in routine diabetes care. Follow‐up with mental health services is suboptimal.

**Keywords:** depression, suicide risk, type 1 diabetes, type 2 diabetes

## Increasing continuous glucose monitoring (CGM) utilization in pediatric type 1 diabetes (T1D) patients with hemoglobin a1c values ≥8.5%

### Ashley Medina^1^, DHSc, Evan Graber ^2,3^, DO, Abigayle Hoover^3^, BS, Kimberly Shoe^2^, APN Patrick Hanley^2,3^, MD, MSHQS

#### ^1^Department of Quality & Safety, Nemours Children's Hospital, Wilmington, DE, USA, ^2^Division of Pediatric Endocrinology, Nemours Children's Hospital, Wilmington, DE, USA, ^3^Sidney Kimmel Medical College of Thomas Jefferson University, Philadelphia, PA, USA

**Background/Objective:** Pediatric T1D patients rarely meet goal hemoglobin A1c (HbA1c) < 7%. CGM utilization can reduce HbA1c by ≥1%. In evaluating causes of elevated HbA1c in our population we noted similar access, but decreased utilization, in patients with HbA1c ≥ 8.5%. Project aims were to investigate reasons for decreased CGM utilization, and to increase CGM utilization in patients with HbA1c ≥8.5% by 10% by July 2025.

**Methods:** Over 3‐months a survey was distributed exploring reasons for not wearing CGM reliably. After collating feedback, we created a tipsheet for patients reviewed by a medical editor, containing QR codes, and available in English and Spanish. The tipsheets were distributed over 4 months and a patient survey eliciting feedback was circulated. A definition for using CGM reliably was established. Documentation of reliable CGM use was standardized in our Diabetes SmartForm (DSF), and a charge for CGM interpretation was generated from the DSF.

**Results:** At baseline, 56% of patients with HbA1c ≥ 8.5% used CGM reliably compared to 82% in patients with HbA1c ≤ 8.5%. The initial survey (*n* = 25) responses centered around challenges with CGM skin adherence. The follow‐up survey responses (*n* = 60) indicated 80% of patients found the tipsheet helpful. Advice on cleaning the skin and keeping the CGM secured with overlay patches and Skin‐Tac were most valuable.

**Conclusions:** This project used patient feedback, a tipsheet, and enhancements in DSF documentation. These interventions increased reliable utilization and documentation of CGM use in patients with T1D and HbA1c ≥ 8.5% from 56% to 76%.

**Keywords:** continuous glucose monitoring, type 1 diabetes mellitus, hemoglobin A1c, diabetes technology

**Figure:**
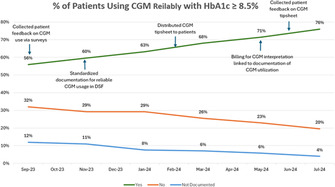
**Figure 1:** Percentage of reliable CGM utilization over time in patients with HbA1c ≥ 8.5%.

## CDCES clinical workflows can address challenges and barriers to equitable care in new onset T1D

### Jeannine Leverenz, Shannon Lin, Anjoli Martinez‐Singh, Barry Conrad, Annette Chiemelski, Ian Chen, Erica Pang, Franziska K Bishop, Priya Prahalad, David M Maahs

**Background/Objective:** The 4T Program (Teamwork, Targets, Technology, and Tight control) at Stanford Children's aims to intensify equitable new‐onset type 1 diabetes (T1D) education to improve outcomes. The Certified Diabetes Care and Education Specialist (CDCES) team has been pivotal in creating standardized workflows to improve access and tailor care to an individual's needs.

**Methods:** Youth with new‐onset T1D start on continuous glucose monitoring (CGM) in the first month of diabetes diagnosis, and a CDCES reviews CGM data monthly. Families are directed to attend a pre‐Automated Insulin Delivery (AID) class between 1 and 3 months post‐T1D diagnosis. To promote equity, the CDCES team created workflows to decrease barriers for families, which include offering diabetes technology to all patients regardless of insurance status or language spoken (Table 1).

**Results:** Since standardizing workflows (Table 1) to encourage patients to complete pre‐AID class in 2022, 79.5% (140/176) of patients completed a pre‐AID class; of those, 37.1% were on public insurance, and 52.9% were minoritized youth (non‐white race and/or Hispanic ethnicity). Pump/AID initiation within 12 months post‐T1D diagnosis was 53.4% (94/176). Overall pump use increased from 49.6% to 53.4%, with AID initiation more than doubling and median time to pump initiation approximately halved from 162 to 85 days with the addition of these standardized workflows in the 4T program.

**Conclusion:** Standardized workflows created by the CDCES team and tailored to patient needs decreased the barriers to technology uptake and increased the use of and shortened initiation time of CGM and AID in the year after T1D diagnosis.

**Keywords:** CDCES, diabetes technology, equity, new onset diabetes

**Table:** Table 1.

**Standardized CDCES Workflows for Equitable New Onset T1D Care**
1. **During Diabetes Self‐Management Education (DSME) at diagnosis, families spend the day with the CDCES and need lunch. The CDCES team works with the social work team to provide food delivery gift cards for the family to order lunch**
2. **CGM and AID systems are introduced by CDCES for anyone with T1D regardless of provider referral, language spoken, and/or insurance status**
3. **Offer pre‐AID classes and trainings in Spanish with our bilingual CDCES**
4. **Offer pump/AID software upgrades in the clinic for youth who may not be comfortable with technology, have inadequate Wi‐Fi, and/or have literacy barriers**
5. **Provide iPod touch devices to families without compatible smart devices for CGM data flow for remote patient monitoring**
6. **Provide CGM supplies for gaps in insurance coverage**
7. **Electronic health record message pool with the CDCES team and study/program coordinators so they can help with any connectivity issues, supply gaps, etc**.

## Transition of care of T1D pediatric population to adult services

### Angela Mojica, MD; Nisha Patel, MD; Katie Fredenburg, RN; Ashley McDuffee, PsyD; Roberto Izquierdo, MD; Jerusha Owusu‐Barnie, MS; Joseph Erardi, BS; Beth Wells, MSN, RN; Jason Sloane, MD

#### ^1^SUNY Upstate Medical University, Syracuse NY, USA

mojicaa@upstate.edu

**Objective:** To have a documented transition plan for 20% of 16‐to‐21‐year olds with type 1 diabetes (T1D) in 12 months, beginning in January 2024, for those enrolled in the program.

**Methods:** Created a set standard curriculum with 12 visits total beginning at age 16. Incorporated nutrition and our behavioral health psychologist into the program by dedicating one visit to each. Created a referral to the program that will be made at age 15 to begin the process to enroll them into the program. Created better tracking within EPIC to track the number of participants in the program, the number of participants with a documented plan, and the number of those completing the program.

**Results:** As of July 2024, 93 individuals, age 16–21, living with T1D have a documented transition plan. With a total population size of 516, the current percent of our T1D population with a documented plan is 18%. Since, the start of 2024, 240 referrals to the transition program have been placed. In 2024, 13 individuals completed the program, of which 9 successfully transitioned to adult endocrinology. In 2024, 32 pediatric patients with T1D successfully completed at least 1 visit at our adult endocrine clinic and 20 of those patients were enrolled in the transition program.

**Conclusions:** Transition of care plans are important because they help those living with diabetes complete all education and gain necessary skills needed to become more independent in managing their care. There is a need to analyze if the standardized curriculum is providing participants with education necessary to manage diabetes in the adult clinic.

**Keywords:** transition of care, pediatrics, document transition plan, type 1 diabetes, referrals

## BRIDGE (barrier reduction in insulin delivery for greater equity) project: increasing insulin pump use in youth with type 1 diabetes with a language of care other than English

### Samantha Goldklang, ARNP, Faisal Malik, MD, MSHS, Yasi Mohsenian, MPH, Joy Briggs, MBA, MSN, CDCES, Meenal Gupta, MD, Alissa J. Roberts, MD

#### Seattle Children's Hospital, Seattle, Washington, USA

Samantha.goldklang@seattlechildrens.org

**Objectives:** The use of insulin pumps for type 1 diabetes (T1D) management in youth is associated with improvement in glycemic outcomes. However, there are significant inequities in diabetes technology use by language of care. The SMART aim of this study is to increase insulin pump use in youth with T1D whose families have a primary language of care other than English (LOE) from 19% to 50% from February 2022 to January 2025.

**Methods:** A key driver diagram was created to identify feasible interventions to increase insulin pump use for families with LOE within the diabetes clinic setting. Implementation of change ideas was followed by Plan‐Do‐Study‐Act cycles to inform ongoing improvement efforts. Interventions included: (1) increasing available languages for pump class, (2) targeted follow up for youth after initiating pump therapy and (3) making educational materials more accessible based on health literacy level and translation to various languages.

**Results**: This study included 165 youth with T1D and LOE with a mean age of 12.4 (4.2 SD) years and mean HbA1c of 10.0 (2.5 SD)%. Most common languages of care other than English were Spanish (53.9%), Somali (10.9%), and Russian (6.7%). Baseline rate of pump use in youth with LOE was 19% in February 2022 and this increased to 36% by June 2024 (Figure 1).

**Conclusions**: Implementation of the BRIDGE project doubled the pump utilization in youth with T1D with LOE over a 2‐year period. Further interventions to assess and address barriers to technology are ongoing.

**Key Words:** Insulin infusion systems, language, pediatrics, Type 1 Diabetes

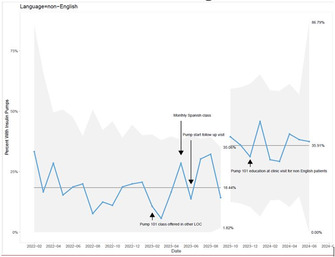

## Implementing high‐risk programs at four pediatric endocrinology clinics in the T1D exchange QI collaborative

### Ori Odugbesan, MD, MPH^1^ ; Trevon Wright, MHA^1^ , Mary Pat Gallagher, MD^2^ ; Jeniece Ilkowitz, BSN, MA, CDCES^2^ ; Selorm A. Dei‐Tutu, MD^3^ ; Campbell, Rebecca^4^; Claire Zimmerman^4^; Roberto Izquierdo MD^5^ ; David Hansen MD^5^ ; Joseph Erardi, BS^5^ ; Osagie Ebekozien MD, MPH^1^

#### ^1^T1D Exchange Boston, MA, USA, ^2^Hassenfeld Children Hospital, NY, USA, ^3^Baylor College of Medicine, TX, USA, ^4^University of Colorado Denver Anschutz Medical Campus, CO, USA, ^5^SUNY Upstate Medical University, Syracuse NY, USA

**Background**: Hemoglobin A1c (HbA1c) levels ≥9% significantly increase the risk of diabetes‐related complications. It is therefore important to implement strategies to lower HbA1c levels among people living with diabetes with persistently elevated HbA1c. This is a multicenter study aimed to evaluate the effectiveness of a multidisciplinary team and patient‐centered approach in reducing HbA1c levels.

**Methodology**: A cohort of people living with diabetes with HbA1c levels ≥9% and managed at one of four diabetes centers were enrolled into high‐risk programs locally. Programs were named to create a positive and supportive initiative, rather than one that labels people as ‘high‐risk’. The centers used a multi‐disciplinary team and patient‐centered approach focused on individualized care plans, education, regular follow‐ups, and tailored interventions to address each person's unique needs. The teams tracked HbA1c levels. Data were collected and shared monthly with the coordinating center.

**Results**: HbA1c levels ≥9% at participating centers decreased by 2.5% from baseline over 17 months. The consistent follow‐up, personalized care plans, including addressing barriers to patient engagement, were key factors contributing to the improvement in HbA1c.

**Conclusion**: A multidisciplinary and patient‐centered approach can effectively help people with elevated HbA1c levels achieve HbA1c levels closer to goal. This approach is feasible and can be successfully implemented in more diabetes care settings.

**Keywords:** multidisciplinary care, patient centered care, HbA1c, High‐risk

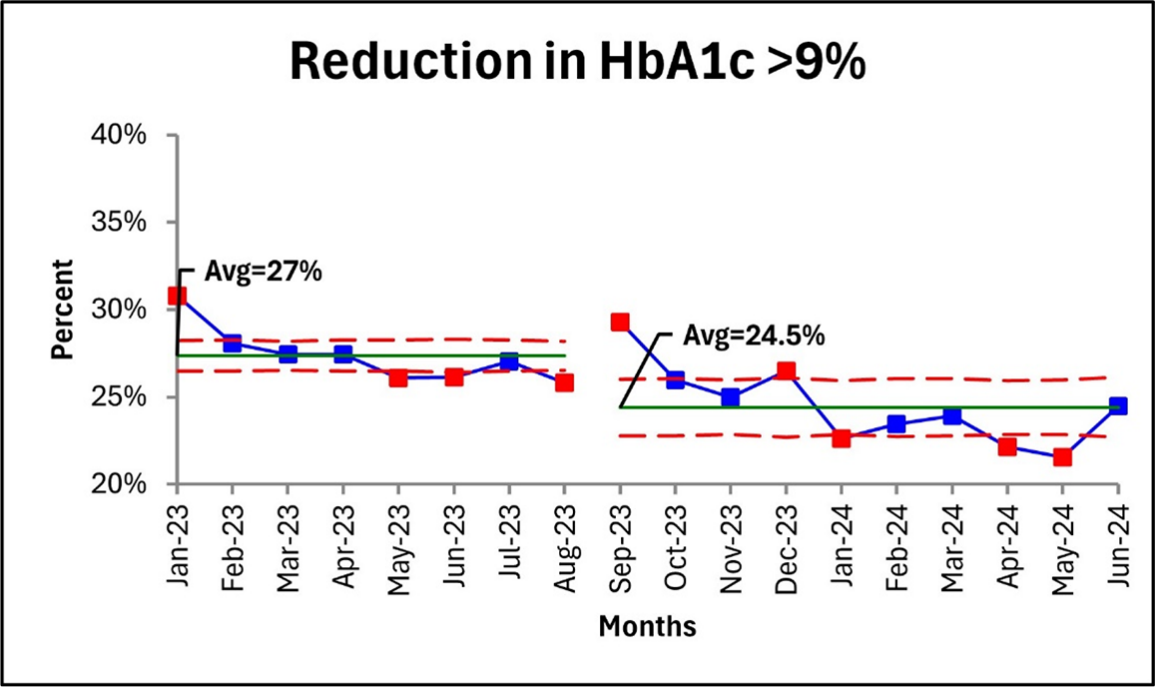

## Diabetes autonomy milestones: Educator and family expectations

### Jessica Schmitt, MD, MSHQS^1^ , Stephanie Simpson CDCES^2^ , Sheila Benton, RN, BSN, CDCES^2^ , Joycelyn Atchison MD^1^ , Christy Foster, MD^1^

#### ^1^University of Alabama at Birmingham Heersink School of Medicine, CPPII M30 1600 7th Ave South Birmingham AL, USA; ^2^Children's of Alabama CPPII M30 1600 7th Ave South Birmingham AL, USA

jessicaschmitt@uabmc.edu

**Background:** Challenges arise when expectations for self‐management in youth with diabetes (YwD) are out of proportion to their developmental stage. We aimed to better understand diabetes educators' approach to education of diabetes autonomy milestones and YwD and their caregiver's beliefs related to age‐appropriate milestones at Children's of Alabama.

**Methods:** To obtain stakeholder input, in March 2024 we surveyed diabetes educators about their approach to education on diabetes autonomy milestones and their needs. In April 2024, a convenience sample of YwD and their caregivers completed a questionnaire about age‐specific diabetes self‐management tasks. Using this feedback, a “Diabetes Autonomy Milestone” tool for caregivers and YwD was created. In June 2024 we tested its use in select clinics.

**Results:** Twelve diabetes educators completed the survey. Half reported having easy access to diabetes milestone educational materials. The most common time educators reviewed milestones was when there were gaps in caregiver expectations and developmental stage of the YwD. (See **Figure 1**). Educators asked for readable concise handouts with specific tasks by age. Seventy‐four caregivers and YwD completed the survey. Twenty‐one percent expected YwD under age 10 to count carbohydrates without supervision. Twenty percent stated a 6‐year‐old with diabetes should be expected to check blood sugar without supervision. Sixteen percent expected children 7 and under to begin learning to give injections.

**Conclusions:** Caregiver and YwD expectations for self‐management are not always in sync with diabetes educator expectations. These results will guide development and evaluation of education tools for age‐appropriate diabetes care tasks.

**Keyworks:** adolescent, child; diabetes mellitus, type 1; self‐management

**Figure:**
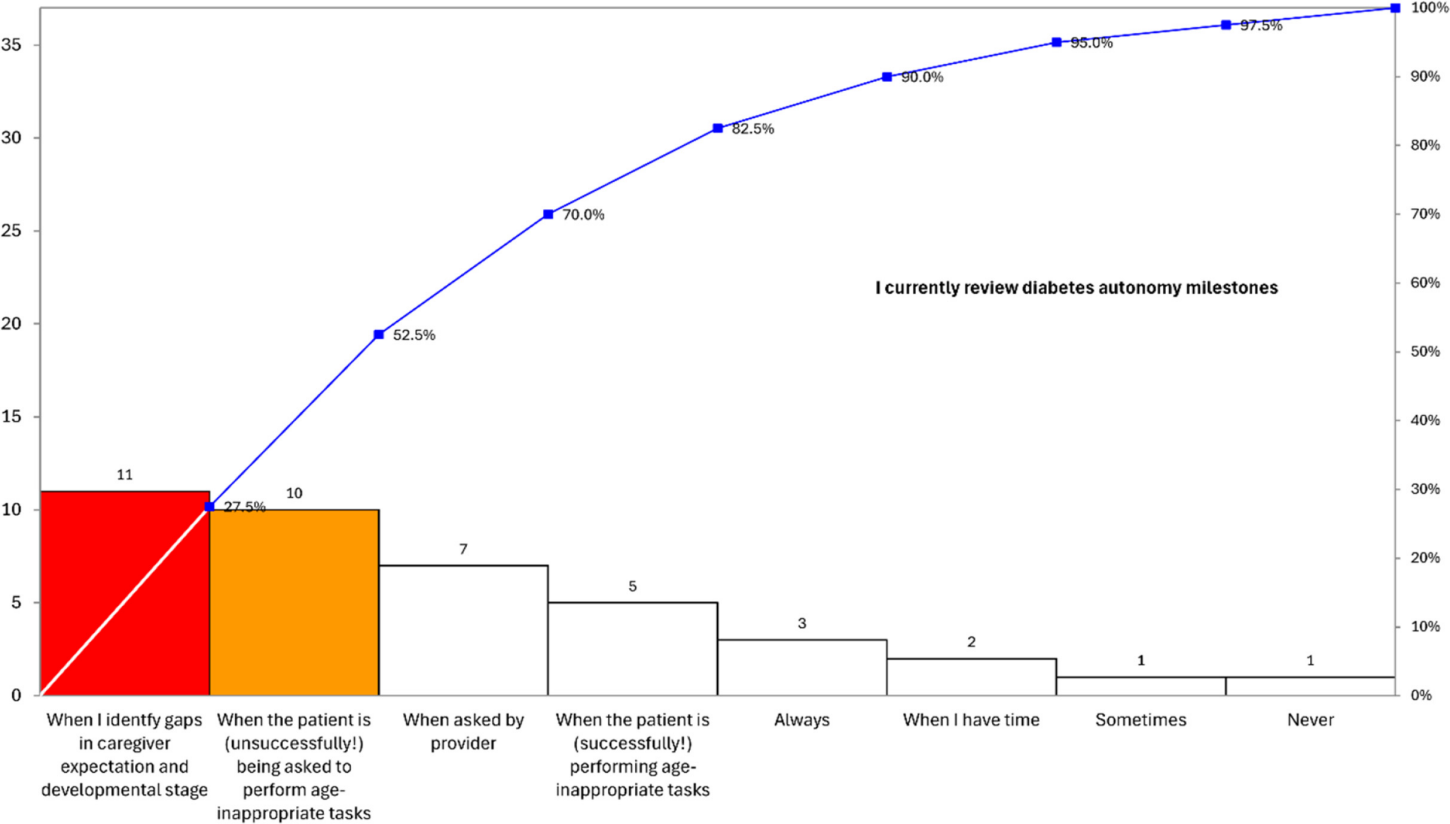
**Figure 1:** Pareto chart of diabetes educators; responses to the question: “I currently review diabetes autonomy milestones…”

## Improving depression screening rates among adolescents with type 1 diabetes using limited clinical resources

### Samantha Jimenez, MD; Stephanie Crossen, MD, MPH; Mia Silva, BS; Amber Lao, CMA; Sarah Woods, NP; Rachael Lee, NP; Stephanie Christensen, MD; Shelby Chen, MD; Nicole Glaser, MD; Caroline Schulmeister, MD

#### University of California, Davis – Division of Pediatric Endocrinology, 2516 Stockton Blvd, Sacramento, CA 95827 USA,

shjimenez@ucdavis.edu

**Background/Objective:** Our clinic has historically struggled to complete annual depression screening among adolescents with type 1 diabetes (T1D) due to limited staff (no psychologist, ¼ social worker). Greater telehealth use post‐COVID‐19 unintentionally worsened screening rates due to restricted time during video visits and competing goals during less frequent in‐person encounters. We therefore initiated a quality improvement project with the aim of increasing our annual depression screening rate to ≥70% for patients with T1D aged 12+.

**Methods:** We created an in‐person annual diabetes (ANDI) visit to enable completion of recommended screening tests. These longer visits occurred in the summer to minimize school absences and weather‐related access barriers. In Fall 2023 we hired a full‐time social worker and created an Epic Smartform to make patients' screening status easily visible to providers. In March 2024 we began giving all patients aged 12+ a paper PHQ‐2 upon check‐in at T1D visits. In Summer 2024 staff were retrained in this process and ANDI visits were restarted.

**Results:** Monthly depression screening rates and plan‐do‐study‐act cycles are shown in Figure 1.

**Conclusion:** Implementation of ANDI visits and improved clinic workflows achieved our aim of ≥70% annual depression screening among adolescents with T1D presenting for care. Dedicated in‐person screening visits and routine PHQ‐2 administration are sustainable and cost‐effective. Next steps include addressing ongoing low screening rates among subpopulations – including non‐English speaking patients and those with neurodevelopmental differences – as well as developing resources to bridge the gap between identification of a positive screen and access to mental health care.

**Keywords:** depression; diabetes mellitus, type 1; mental health services

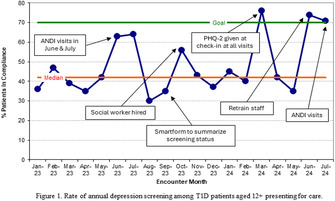

## Increasing lipid profile screening in youth with type 2 diabetes

### Puja Singh^1,2^, MD; Christy Byer‐Mendoza^2^, MSN, RN, CNS, CPN, CDCES; Kim McNamara^2^ , RN, BSN, CDCES; Andrea Huber^2^, RN, BSN, CDCES; Jennifer Ruiz^2^, BSN, RN, CPN; Mario Bialostozky^1,2^, MD; Carla Demeterco‐Berggren^1,2^, MD, PhD

#### ^1^University of California San Diego, San Diego, CA, USA, ^2^Rady Children's Hospital San Diego, San Diego, CA, USA

pusingh@health.ucsd.edu

**Background/Objective:** Type 2 Diabetes (T2D) in youth has been shown to be a more rapidly progressive disease and patients may have evidence of microvascular complications at time of diagnosis. As such, lipid screening is crucial for early detection of abnormal cholesterol levels to prevent future adverse cardiovascular outcomes. The aim of our QI project was to increase the percentage of patients with T2D who had lipid profile performed in the last 12 months from a baseline of 70%–90% by May 31, 2024.

**Methods:** Using a diabetes dashboard within the electronic health record (EHR), we were able to identify the patients with T2D who were overdue for lipid panel screening prior to their upcoming clinic appointments on a weekly basis. A multidisciplinary team identified key areas for improvement and interventions were tested through multiple PDSA cycles. Interventions included healthcare provider education on American Diabetes Association (ADA) recommendations, creation of an educational handout for patients and caregivers, health maintenance reminders within EHR on patients overdue for lipid screening, and implementation of a point of care (POCT) lipid machine in the diabetes clinic.

**Results:** As of May 31, 2024, the percentage of patients with T2D who had lipid profile completed reached the target of 90% (Figure 1).

**Conclusion:** Implementation of QI methodology led to improvement in lipid profile completion in youth with T2D. Utilizing EMR tools, weekly data collection, and POCT machine helped standardize lipid screening which will lead to early recognition of microvascular complications and improved health outcomes.

**Keywords**: type 2 diabetes, pediatrics, lipid screening, quality improvement

**Figure:**
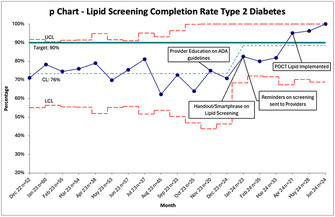
**Figure 1**: Percentage of youth with type 2 diabetes with lipid profile screening completion.

## Insulin cost and rationing in the pediatric type 1 diabetes population

### Lauren Waterman, MD^1^ ; Erin Cobry, MD^1^ ; Casey Sakamoto, MS^2^ ; Talia Thompson, PhD^3^ ; Sarit Polsky, MD, MPH^1^ ; Katherine Berrian, BS^1^ ; G. Todd Alonso, MD^1^

#### ^1^University of Colorado Anschutz Medical Campus, Barbara Davis Center for Diabetes, Aurora, CO, USA; ^2^Colorado School of Public Health, Department of Biostatistics and Informatics, Aurora CO, USA; ^3^Univestiy of Colorado Anschutz Medical Campus, Department of Pediatrics, Aurora, CO, USA

lauren.waterman@cuanschutz.edu

**Background/Objective:** Little is known about the impact of high insulin costs on youth with type 1 diabetes (T1D) in the United States. We designed a survey to identify gaps and opportunities for improvement in clinician awareness of patient affordability of insulin.

**Methods:** Patient/parent‐reported frequency of insulin rationing behaviors, cost concerns, and most recent insulin out‐of‐pocket cost was assessed by a survey developed at the Barbara Davis Center. Demographics, insurance status, hemoglobin A1c [HbA1c], and Time in Range [TIR] were obtained from medical record review. Linear models were fit to compare out‐of‐pocket costs, HbA1c, and TIR, adjusting for sex, diabetes duration, race/ethnicity, insurance, and age.

**Results:** Of 146 respondents (patient age 14.9 ± 4.8 years, diabetes duration 6.7 ± 4.7 years, 87.7% non‐Hispanic White [NHW], 79.5% private insurance), 22.6% (33/146) reported running out of insulin before next refill and 15.8% (23/146) reported using less insulin than needed to prolong supplies (Table). People who reported rationing behaviors (*N* = 50, 16.1 ± 5.2 years old, diabetes duration 7.8 ± 5.2 years, 82.0% NHW, 74.0% private insurance) had higher monthly insulin costs ($77.60 v. $19.30, *p* = 0.004), lower TIR (−9.78 ± 4.03%, *p* = 0.02) and higher HbA1c (+0.69 ± 0.23%, *p* = 0.003) compared to those who denied rationing.

**Conclusions:** Insulin rationing was common among youth with T1D and was associated with paying higher monthly insulin costs and higher glycemia. More than 10% of respondents reported not filling an insulin prescription or skipping paying other bills due to insulin cost. Future work will introduce routine screening for insulin rationing and improved methods to educate patients about insulin access programs.

**Keywords:** healthcare costs, healthcare rationing, type 1 diabetes

**Table:** Table 1. Patient / parent responses to insulin access survey.

Survey question	Agreement (%)
In the last year, have you run out of insulin before you are allowed to have another refill	22.6
In the last year, have you not filled a prescription for insulin because you could not pay for it	11.6
In the last year, have you used less insulin than you needed to avoid running out	15.8
Insulin was too expensive for me to afford in the last year	21.5
I am worried that I will not be able to afford insulin in the next 3 months	19.8
I have skipped paying other bills (groceries, rent, utilities) to afford my insulin	12.3
I know what to do if the insulin price is more than I expected	61.6
I know who to contact at clinic if I cannot afford my insulin	54.8
I feel comfortable discussing with my provider or team if I am worried, I will not be able to pay for insulin	80.8

## Implementation of type 1 diabetes transition clinic and visit checklist

### Jordan D. Ross, MD; Blake Adams, BSN^2^ ; Kayla Zimmerman, BA, BS; Grace Nelson, MD; Anne Wynn, MD^3^

#### 1.University of Tennessee Health Science Center Memphis, Tennessee, USA; 2. Le Bonheur Children's Hospital Memphis, Tennessee, USA; 3. Regional One Health Memphis, Tennessee, USA

jross27@uthsc.edu

**Background/Objective:** Comprehensive type 1 diabetes transition clinics improve adolescents' satisfaction with their medical care and limit lapses in care during this vulnerable period. Our team started a T1D transition clinic visit at Le Bonheur Children's Hospital in 2018. We updated our program to allow for a larger capacity and to track outcomes and standards more quantitatively.

**Methods:** Our team increased the T1D transition clinic from two clinics to four clinics per month in September 2023 to expand capacity. We designed and implemented a checklist of important medical and psychosocial topics to follow patients through all their transition clinic visits. From July 1, 2023, to June 30, 2024, all diabetes clinic encounters were pulled for patients with T1D and at least 16 years of age.

**Results:** Of the 735 diabetes clinic encounters for 241 unique adolescent patients, 29.5% (*n* = 71) attended T1D transition clinic at least once. Of these 71 attendees, 29 (41% of attendees) had at least two encounters. The transition clinic visit checklist was completed with 37.5% (*n* = 6) of the 16 patients seen in transition clinic after its implementation in July 2024.

**Conclusions:** Expanding the number of transition clinics allows more adolescents to complete a T1D transition program. Nationally, formal definitions of these programs are emerging. As part of these standards, a transition clinic visit checklist (ideally an editable form in the electronic medical record) ensures that important topics are addressed with each adolescent at an individualized pace.

**Keywords**: checklist; diabetes mellitus, type 1; transition to adult care

## Improving prescribing rates of GLP1 receptor agonists (GLP1‐RA) in youth with type 2 diabetes

### Alyssa Huang, MD, Alissa J. Roberts, MD, Yasi Mohsenian, MPH, Grace Kim, MD

#### University of Washington, Department of Peds, Seattle, Washington, USA

alyssa.huang@seattlechildrens.org

**Objectives:** Youth onset type 2 diabetes (T2D) is becoming increasingly prevalent and is an aggressive disease leading to early failure requiring insulin therapy and early comorbidities compared to adult onset T2D. Thus, youth with T2D should pursue aggressive therapy and aim to achieve a lower A1C target to prevent diabetes related complications. GLP1 receptor agonists (GLP1‐RA) were FDA approved in June 2019 for the treatment of youth T2D; however, the confidence of initiating GLP‐1RA was low in our clinic. We sought to improve prescribing rates of GLP1‐ RA therapy in our youth with T2D.

**Methods:** We developed education about GLP1‐RA for our medical providers and staff including a presentation and handouts. Our team also partnered with nursing to implement an insulin weaning protocol that allowed nurses to independently help titrate GLP1‐RA and insulin doses in youth with T2D.

**Results:** After implementation of our quality improvement educational intervention, the mean monthly prescribing rate for GLP1 therapy increased from 7% of patients with T2D to 12% after 1 year post intervention. The mean HbA1c was similar for all patients with T2D prior to the intervention compared to 1 year after the intervention (7.8 ± 2.3% vs. 7.8 ± 2.7%).

**Conclusions:** Providing education on GLP1‐RA and partnering with nursing staff to titrate GLP1‐RA through an insulin wean protocol helped increase the prescription rates of GLP‐RA in our youth with T2D. Future work aims to identify barriers that patients may face obtaining GLP1‐RA therapy, effects on weight and streamlining nursing staff implementation of the insulin weaning protocol.

## Standardizing inpatient nursing diabetes education

### Elizabeth Gunckle, MSN, RN, CPNP‐PC; Emily Coppedge, MSN, RN, CPNP‐PC, CDCES

#### Weill Cornell Medicine, New York, NY, USA

elg4011@med.cornell.edu

**Background/Objective:** Pediatric patients and families with newly diagnosed Type 1 Diabetes (T1D) require significant education. A survey of nurses, the key drivers of inpatient diabetes education, revealed various knowledge gaps. We hypothesized that targeted education by the pediatric diabetes team would help improve inpatient nurses' comfort in delivering patient education in these areas.

**Methods:** A Qualtrics survey was developed and distributed to inpatient pediatric nurses to assess comfort levels for diabetes topics (1 = very uncomfortable to 5 = very comfortable). Identified areas of concern included carb counting, insulin dosing, and providing general diabetes education. We created three 1‐hour sessions to provide comprehensive diabetes education, focusing on these topics. Our carb counting and insulin dosing session involved 11 interactive polls to apply concepts and assess for knowledge gaps. A post‐intervention Qualtrics survey was administered 2 weeks following the final session.

**Results:** Eleven participants completed the initial survey, and four participants completed the post survey. The average comfort level for questions about carb counting increased from 4.09/5 to 4.5/5. The average comfort level for providing general diabetes education and answering questions about insulin both increased from 4.27/5 to 4.75/5.

**Conclusions:** Focused teaching increases nursing comfort in providing inpatient diabetes education. We advocate for training the hospital nursing educator to incorporate these sessions into new nursing orientation along with offering quarterly refresher classes, with potential to include other disciplines in the future.

**Key Words:** Diabetes Mellitus Type 1, Pediatrics, nursing education

## Pediatric new onset type 2 diabetes outpatient management

### Monica E. Bianco, MD; Sean DeLacey, MD; Naomi Fogel, MD; Laura Levin, DO; Jiarui Li, MS; and Paula Petrie, RN

#### Ann & Robert H. Lurie Children's Hospital of Chicago, Chicago, Il/USA

mbianco@luriechildrens.org

**Background/Objective:** Pediatric patients with new onset type 2 diabetes (T2D) with a hemoglobin A1c (HbA1c) ≥9% are typically admitted for diabetes management and education. Our goal is to transition to outpatient management to decrease the burden on families and our health system. A protocol was developed to aid this transition in management.

**Methods:** The protocol for the outpatient management of new onset T2D was developed in collaboration the diabetes team and implemented in June 2024. Using the Electronic Medical Record, we performed a retrospective review of patients with new onset T2D with HbA1c ≥9% diagnosed 6/1/2023–5/1/2024 who received inpatient management and will prospectively monitor those treated in the outpatient setting from 6/1/2024 to 12/31/2024. The HbA1c at 3 months post‐diagnosis between the two groups will be compared to assess for short‐term impact on outcomes.

**Results:** Prior to implementation, 0–4 patients per month were diagnosed with new onset T2D and a HbA1c ≥9% and were admitted. Average length of stay was 2.3 days. Prior to implementation, the HbA1C at 3 months after diagnosis for patients with HbA1c ≥9% was 7.6% and for HbA1c <9% was 6.2%. After implementation, in June of 2024, two patients presented with T2D diagnosis and a HbA1c ≥9% and both were treated outpatient.

**Conclusions:** Treating patients with new onset T2D and a HbA1c ≥9% in an outpatient setting is feasible at our institution. It will take time to assess the impact on short‐term glycemic outcomes after this change in management.

**Keywords**: Inpatient, outpatient, pediatrics, type 2 diabetes mellitus

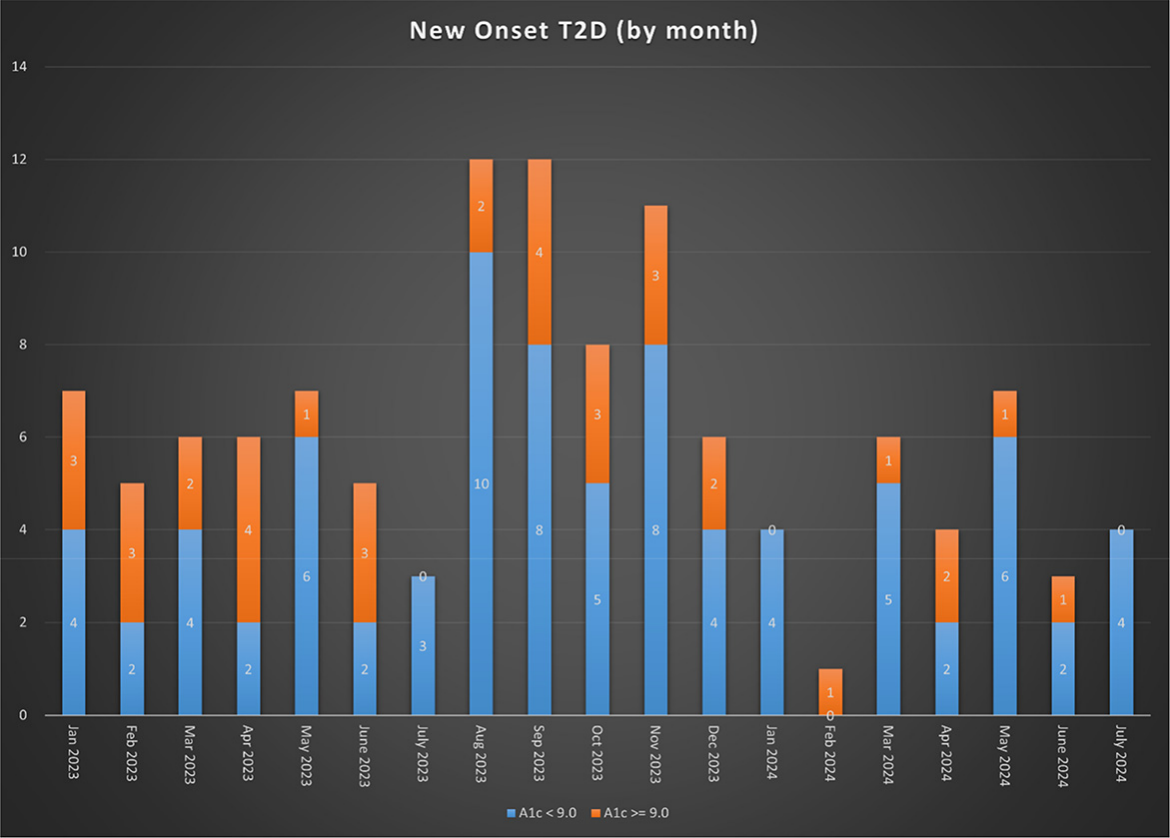

## Insulin pump DKA prevention

### Paula Petrie, BSN, RN, CDCES and Naomi R. Fogel, MD

#### Ann & Robert H. Lurie Children's Hospital of Chicago, Chicago, IL, USA

pipetrie@luriechildrens.org

**Background/Objective:** Emergency department and hospital admissions related to insulin pump failure are often due to lack of timely intervention at home. The aim of the project is to decrease the average monthly number of emergency department and hospital admissions among patients on insulin pump by 20% within 1 year of introduction of a printed patient teaching tool.

**Methods:** Electronic medical record data was used to measure the average number of patients on pump admitted to the emergency department and hospital per month. Included were patients followed by Lurie Children's. Excluded were patients with repeat admissions or admissions not related to pump mismanagement. A patient teaching tool was developed with expert input from the interdisciplinary diabetes team at Lurie Children's Hospital and utilized with patients on pump in the hospital and outpatient settings.

**Results:** Baseline admissions averaged 6 per month the 7 months prior to the project start. In the 9 months since the project announcement, admissions have averaged 2 per month, representing a 66% decrease from baseline.

**Conclusions:** The decrease in admissions coincided with the project announcement to the team, not necessarily with the start of the use of the tool with patient families. Raising awareness of the issue to the clinical staff may have generated more informal discussion with patients thus increasing timely intervention at home and preventing admissions. Further iterations of the teaching tool are underway, including incorporation into the electronic medical record.

**Keywords:** diabetes mellitus, diabetic ketoacidosis, insulin infusion systems, insulin pump

## Risk factors for CGM non‐use

### Naomi R. Fogel, MD; Anna Wood, MPH; Shan Sun, PhD

#### Ann & Robert H. Lurie Children's Hospital of Chicago, Chicago, IL, USA

NFogel@luriechildrens.org

**Background/Objective:** Continuous glucose monitor (CGM) use in Type 1 Diabetes improves glycemic outcomes and reduces hypoglycemia. CGM prescription at diagnosis and discussion at clinic visits increases the percentage of patients with active prescriptions. However, barriers remain to consistent use over 70% of the time. We aim to identify risk factors for CGM non‐use to inform future interventions.

**Methods:** From January 1 to December 31, 2023, data were collected on patient demographics, CGM use and wear time at each outpatient diabetes visit. Descriptive statistics, bivariate analyses, and multivariable logistic regression using a backward elimination model selection method were performed to identify potential risk factors for CGM non‐use.

**Results:** The most significant risk factor for CGM non‐use was the Child Opportunity Index (COI), which uses indicators based on zip code of residence. In the multivariable model, after controlling for patient sex, age, race and ethnicity, language, insurance, having a complex chronic condition, visit department, visit provider, insulin regimen, MyChart activation, whether they sent MyChart message(s), and appointment completion rate ≥75%, patients with “Very Low” and “Low” COI had higher odds of CGM non‐use compared to patients with a “Very High” COI (adjusted odds ratio, 8.83; 95% confidence interval, 4.34–17.95 and adjusted odds ratio, 2.93; 95% confidence interval, 1.38–6.21; respectively) (Figure 1).

**Conclusions:** Children with a “Very Low” and “Low” COI are significantly less likely to use their CGM consistently. Future interventions, such as a mobile health van with technology support, will be targeted to this population.

**Keywords:** healthcare disparities, health technology, glucose monitoring, type 1 diabetes

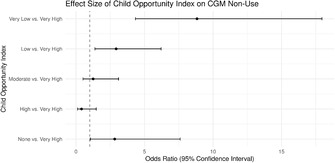

## Improving engagement with annual electronic psychosocial screening among youth with T1D

### Kelsey R. Howard, PhD;^1,2^ Monica Bianco, MD;^1,2^; Sean DeLacey, MD;^1,2^ Laura Levin, DO;^1,2^ Mary McCauley, MD;^1,2^ Jillian Merrick, PhD;^1^ Paula Petrie, RN, CDCES;^1^ Wendy Perez, MHA;^1^ Mayra Ramos, MS;^1^ Jill Weissberg‐Benchell, PhD, CDCES;^1,2^ Naomi Fogel, MD^1^ ^,2^

#### Ann & Robert H. Lurie Children's Hospital of Chicago, Northwestern University Feinberg School of Medicine, Chicago, IL, USA

kehoward@luriechildrens.org

**Background/Objective:** Annual psychosocial screening is recommended for youth with Type 1 Diabetes (T1D); however, successful implementation of screening can be challenging. The objective of this study was to evaluate survey completion rates before and after quality improvement (QI) initiatives intended to reduce patient questionnaire burden.

**Methods:** QI process completed in 2022–2023 to improve the annual psychosocial screening process for patients with T1D followed in the diabetes program at an academically‐oriented children's hospital. Planning revealed workflow challenges were among existing barriers. An intervention of simplifying the psychosocial questionnaire battery to two instruments (depression and diabetes distress) was established. Patients were invited to complete annual psychosocial questionnaires via MyChart. We examined rates of psychosocial screening survey completion during two 9‐month periods before and after the implementation of QI intervention.

**Results:** Prior to intervention, a total of 478 patients were assigned psychosocial screening instruments during a nine‐month period. Of those, 40.17% (*n* = 192) completed at least one screening instrument. In the nine‐month period following QI initiatives, a total of 543 patients were assigned psychosocial screening instruments. Of those, 45.89% (*n* = 249) completed at least one screening instrument.

**Conclusions:** Following interventions to reduce questionnaire burden, we observed an increased number of patients identified for survey completion as well as an increased rate of completion. Additional barriers were identified during QI processes related to technology, social/psychological factors, and messaging. Work is needed to address remaining challenges.

**Keywords:** diabetes mellitus, type 1; depression; quality improvement

## Barriers to achieving diabetes device equity and access for minority patients

### Emma Mason, BS; H. Kaan Akturk, MD

#### Barbara Davis Center for Diabetes, Denver, CO, USA

emma.mason@cuanschutz.edu

**Background/Objective:** Access to diabetes technologies is essential to improving results and meeting diabetes care goals. However, there is a significant technology use gap between non‐Hispanic white patients and minority patients. We examined the technology rates for different races and sought to determine what barriers patients face to getting diabetes technologies.

**Methods:** We identified minority patients, seen in the past year, not meeting diabetes care goals, and not using diabetes technology. We tracked patient attendance at their scheduled appointments, and whether or not they showed up.

**Results:** Between December 2023 and March 2024, we identified 99 Hispanic and non‐Hispanic Black patients who were not using technology and not meeting diabetes care goals. 33 of these patients did not have an appointment scheduled. 28 of these patients did not show up to their appointment or their appointment was canceled. Therefore, nearly 62% of minority patients not meeting their diabetes goals and not using diabetes technology, did not have an appointment scheduled, or did not show up to their routine diabetes care appointment.

**Conclusions:** To increase the amount of minority patients using diabetes devices, patients need to be seen by a diabetes provider. While there are likely other barriers that may need to be addressed to increase device uptake in this population, these patients need to attend routine endocrinology visits to increase device use in this population and therefore have better health outcomes.

**Keywords:** endocrinology, health equity, quality improvement, type 1 diabetes

## Update: pre‐visit diabetes device data capture

### Edelina Cohen, MS RD; Michael Natter, MD; Lauren Golden, MD

#### Center for Diabetes & Metabolic Health at NYU Langone Health New York, NY, United States

edelina.bustamante@nyulangone.org

**Background/Objective:** Diabetes device data capture is essential for effective and efficient patient care. Our practice continues to aim to improve the pre‐visit downloads/uploads (“data”) prior to patient visit with provider from baseline.

**Methods:** An assigned secretary or MA (staff) collects the diabetes data prior to patient visits from June 2023–May 2024. A 5‐day notice was sent to patients via MyChart with instructions on how to download their diabetes device (continuous glucose monitor, meter, insulin pump, or smart pen) reports and upload as an attachment on MyChart, if not already linked to share their data with our practice. The staff placed a follow up call 24–48 hours prior to the patient's visit to remind them to download/upload their diabetes device data or link their device with the practice.

**Results:** After initial intervention, data capture averaged 52% over 24 weeks. Follow up data capture for 4 weeks in Jun 2023 averaged 46%, 2 weeks in Nov 2023 averaged 48%, and 2 weeks in Apr–May 2024 averaged 45%.

**Conclusions:** Follow up diabetes device data capture (averaging 46%) decreased from initial intervention by 12% likely due to staffing. Future steps to improve diabetes device data capture: adequate staffing and standardized diabetes data collection by staff.

**Keywords:** diabetes, device, insulin, glucose

**Table:** Table 1. Percentage of diabetes device data captured prior to visit.

Diabetes Device Data Capture PRE‐visit	% Average
Baseline: Nov to mid Apr 2023 (24 weeks)	52
June 2023 (4 weeks)	46
Nov 2023 (2 weeks)	48
Apr‐ May 2024 (2 weeks)	45

## Insulin pump training or upgrade to visit with provider

### Edelina Cohen, RD; Lauren Golden, MD

#### Center for Diabetes & Metabolic Health at NYU Langone Health New York, NY, USA

edelina.bustamante@nyulangone.org

**Background/Objective:** Frequent contact with the diabetes team in the initial weeks after insulin pump training is essential to optimize insulin pump settings and address pump‐related concerns. Our practice identified a deficit in post pump training or upgrade visit with provider, so we aimed to increase the number of visits within 2–3 weeks of patient's hybrid closed loop (HCL) insulin pump training or software upgrade by 10%–15% by May 2024.

**Methods:** Provider engages in shared decision making with patient to discuss various (HCL) insulin pump options. RN and/or secretary are notified to start insulin pump order process. Diabetes team tracks the patient order, pump training date, and 2–3 week follow up appointment with provider on Excel spreadsheet (tracker). Data is compared to baseline average number of days from pump training (or upgrade) to visit with provider.

**Results:** Based on random 10 patients from tracker, the average number of days from pump training to visit with provider at baseline = 53.6 days, Aug‐Nov 2023 = 22.6 days, and Jan‐Jun 2024 = 21.9 days.

**Conclusions:** A 41% increase in average days from new pump training or upgrade to visit with provider was observed after intervention. This is attributed to ongoing improvement in insulin pump order process and tracking.

**Keywords:** diabetes, insulin, pump, device

**Table:** **Table 1**. Data: average number of days from pump training or upgrade to visit with provider.

	Baseline	Aug–Nov 2023	Jan–Jun 2024
N	10	10	10
% male	50	20	30
% female	50	80	70
% Diabetes Type 1	100	90	100
% Diabetes Type 2	0	10	0
Average # days from pump training (or upgrade) to visit with provider	53.6	22.6	21.9

## Updating ketone action plan for automated insulin delivery pumps: children's healthcare of Atlanta

### Catherine Rust MS, RD, LD, CDCES^1^ ; Kristina Cossen MD^1^

#### ^1^Children's Healthcare of Atlanta

**Background:** Automated insulin delivery systems (AID) use algorithms from continuous glucose monitor input to adjust insulin administration. However, this complicates the management of ketones during pump failure as these algorithms cannot account for on board subcutaneous insulin which is standard practice for ketone management in pump failure. This project aimed to increase knowledge and recall by 10% with a new AID ketone action plan (KAP) in a pediatric diabetes clinic from December 2023 to April 2024.

**Methods:** Children's Healthcare of Atlanta created a 5 question post‐teaching survey for families to complete. This survey was provided to 12 families at baseline and 13 families after the implementation of the updated KAP. Multiple Plan‐Do‐Study‐Act (PDSA) cycles were performed and included: creation of KAP, creation of visual representation of KAP, in‐person education sessions, and follow up questionnaires.

**Results:** Out of the 12 families evaluated at baseline, six responded to the survey with an average score of 47% for correct answers. After education using the updated KAP, 13 families were evaluated, and five of them responded, with an improved average score of 56% correct answers. The magnet contributed to better responses for 3 out of the 5 questions.

**Conclusions:** Ketone management on pump therapy can be burdensome. The use of KAP is effective and feasible to improve knowledge and recall. The team will include the visual representation of the KAP in a mobile app through CHOA.

**Keywords**: sick day management, ketones, automated insulin delivery systems.

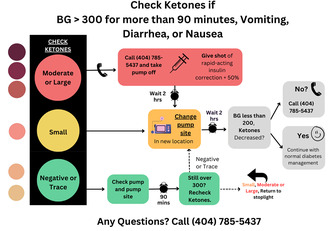

## Implementation of psychosocial support interventions to narrow equity gaps in T1D outcomes: ConnecT1D quality improvement (QI) initiative

### Laura Smith, PhD, CDCES; Sarah Corathers, MD, Molly Williams, MSW; Amy Grant, DNP, RN, CPN; Marissa Town, BSN, RN, CDCES; Amanda Riley, MS, RD, LD, CDCES; Amanda Howell, MPH, CPH; Nana‐Hawa Yayah Jones, MD

#### Cincinnati Children's Hospital Medical Center, University of Cincinnati College of Medicine, Cincinnati, OH, USA

Laura.Smith4@cchmc.org

**Background/Objective:** Despite advancements in diabetes technology, managing type 1 diabetes (T1D) remains challenging, with notable gaps between best practice guidelines and the provision of psychosocial support. ConnecT1D, a comprehensive quality improvement (QI) project, addresses these challenges to enhance mental health and social functioning in T1D management by embedding social work (SW) and psychology within a specialized clinic.

**Methods:** We implemented a multidisciplinary diabetes clinic restricted to youth at risk for diabetes complications identified from social determinants of health (SDoH) screenings, frequent hospitalizations, lack of diabetes technology, and publicly insured. A clinical psychologist and SW were embedded into these clinics. Rates of SW/psychology visits were tracked using statistical process control (SPC) charts.

**Results:** Rates of annual SW/psychology visits increased from 67% to 77%, over 3 years for youth with T1D on public insurance (see Figure 1). Compared to youth who were not seen in a clinical encounter with an embedded SW or psychologist, youth who were seen were more likely to have reported an SDoH, consistent clinic visits, a mental health diagnosis, and a recent hospitalization. Youth seen by SW had higher A1c values (8.9% for those seen versus 8.2% for those not seen) while youth seen by the psychologist had higher rates of missed appointments.

**Conclusions:** Youth with T1D on public insurance evidenced an increase in SW/psychology visits during the implementation of the ConnecT1D project which included multi‐faceted psychosocial intervention strategies aimed at providing in clinic support for patients and families, thereby reducing disparities in psychosocial support interventions.

**Key Words:** Diabetes Mellitus, Type 1; Social Determinants of Health, Health Equity

**Figure:**
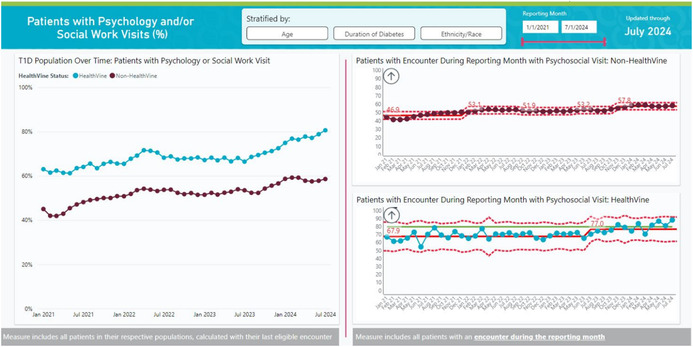
**Figure 1:** Rates of annual SW/psychology visits over time stratified by public insurance status. Public insurance in blue (Healthvine) and private insurance in maroon. Both cohorts experienced an increase of 10% over baseline visit rate.

## Increasing equitable access in diabetes tech among patients with type 1 diabetes across a health system

### Suma Gondi, MD; Paige Dixon, MD; Madeleine Rouviere, RD; Camilla Levister, NP; Nirali Shah, MD; Carol Levy, MD; Grenye O'Malley, MD

#### Mount Sinai Health System, New York, New York, USA

suma.gondi@mssm.edu

**Objective**: Within our urban academic health system, some sites integrate all payors while others have different practices for private versus Medicaid insurance. Provider staffing is similar across sites but ancillary staff differs. We aim to compare technology uptake between two clinic models ‐ combined versus split insurance ‐ and track responses to quality improvement interventions.

**Methods:** Epic reports identified pump, continuous glucose monitors (CGM), and smart insulin pen (SIP) users based on diagnosis codes, problem lists, and CPT/medication orders from July 2023 to July 2024. Provider surveys and semi‐structured patient interviews identified barriers to usage.

**Results:** Comparing combined vs. split clinics (Table 1): CGM use was 86% versus 78% for Medicaid, 60 versus 57% for Medicare, and 92 versus 82% for commercial insurance; pump was 61% versus 41% for Medicaid, 30% versus 27% for Medicare, and 64% versus 55% for commercial insurance. SIP usage was minimal from 0% to 4%. Provider barriers included unfamiliarity and cumbersome prescribing processes. Patients reported technology was often not discussed due to time constraints and inconsistent provider continuity.

**Conclusion:** Usage differed between sites most drastically for pumps for Medicaid patients and was lowest at both sites for Medicare patients. Addressing identified barriers could improve usage and address this inequity in access. We will implement interventions using the Plan, Do, Study, Act quality improvement model, including new prescribing smartphrases, standardized educator visits, and automated outreach to non‐tech users to address bias and lack of continuity.

**Keywords:** diabetes mellitus, type 1; equity; healthcare delivery; insulin infusion systems; quality improvement

**Table:** **Table 1:** Diabetes technology usage by clinic site and insurance type.

	Combined (*n* = 378)	Split (*n* = 953)
CGM		
Medicaid	86% (114/132)	78% (81/104)
Medicare	68% (36/53)	57% (92/161)
Commercial	92% (178/193)	82% (564/688)
Pumps		
Medicaid	61% (80/132)	41% (43/104)
Medicare	30% (16/53)	27% (44/161)
Commercial	64% (123/193)	55% (378/688)
SIP		
Medicaid	2% (2/132)	4% (3/104)
Medicare	2% (1/53)	1% (1/161)
Commercial	2% (3/193)	2% (10/688)

## Insulin delivery devices in management of T1D among pediatric patients

### Jennifer Navarro, MD^1^ ; Tossaporn Seeherunvong, MD^1^ ; Patricia Gomez, MD^1^ ; Ori Odugbesan, MD, MPH^2^ ; Janine Sanchez, MD^1^

#### ^1^Jackson Medical Health System/ University of Miami, Miami, FL, USA, ^2^ T1D Exchange, Boston, USA

Jennifer.navarro@jhsmiami.org

**Background/Objective:** Our study aimed to evaluate the different insulin delivery devices and their impact on A1c outcomes within our patient population.

**Methods:** Data was acquired via chart review on all clinic patients with T1D seen in 2023, overall, 837 patient encounters by 3 physicians. We compared A1C and insulin delivery methods: multiple daily injections (MDI), smart pen (SP), non‐hybrid pumps (NHP), and hybrid pumps (HP).

**Results:** Overall, 44% of patients used HPs, 8% used NHPs, 24% used SP, and 25% on MDI. The use of devices varied by providers. For provider 1, the median A1C values were 7.4% for HPs, 7.2% for NHPs, 8.6% for SP, and 8.15% for MDI. Among HP users, 54.76% achieved A1C < 7.5%. For provider 2, the median A1C was 7.6% for HPs, 8.1% for NHPs, 10.1% for SP, and 9.3% for MDI. In this group, 34% of HP users had A1C < 7.5%. For provider 3, the median A1C was 7.0% for HPs, 7.1% for NHPs, 8.0% for SP, and 7.6% for MDI. Here, 71.03% of HP users achieved A1C < 7.5%. Provider 2's patients were mostly publicly insured.

**Conclusions:** The highest median A1C was among SP users, followed by MDI users. HP users had the lowest median A1C and the highest percentage of A1C < 7.5%, while SP users had the lowest percentage. Results may be affected by patient preference for insulin delivery devices and correct use of the device. These findings indicate that hybrid pumps help more effectively lower A1C.

**Keywords:** insulin delivery, hybrid pump, non‐hybrid pump, smart pen, multiple daily injections

## Improving clinic visits for adolescents with HbA1c ≥ 8

### Jessica Landau, D.O., Karen Chen M.D., Janine Sanchez M.D., Tossaporn Seeherunvong M.D., Patricia Gomez M.D.

#### Jackson Memorial Hospital, Holtz Children's Hospital, University of Miami Pediatric Endocrinology, Miami, Florida, USA

jessica.landau@jhsmiami.org, karen.chenchen@jhsmiami.org

**Background/Objective:** To better understand how to improve clinic visits for adolescents with T1D with A1c ≥ 8.

**Method:** We created a Likert scale survey to assess understanding of T1D, perceived benefits of clinic visits, and areas for clinician improvement. The initial pilot study was completed by 15 patients. Adolescents (aged 12–19) with an A1c ≥ 10 who had been diagnosed for >1 year were offered the survey at their clinic visit. With the valuable input of the People with Diabetes Advisory Committee from the T1DX we refined our survey language. We have also expanded to include those with A1c ≥ 8. The results were de‐identified.

**Results:** Surveys collected demonstrate that 49% “agree” that they are managing their diabetes well, however, 50% of the patients “agree” with being concerned about their diabetes, and 14% “strongly agree.” More than 60% of patients agreed that hearing about the consequences of diabetes motivates them to use insulin and check their glucose more frequently. However, 30% of patients reported feeling discouraged when hearing about the consequences of diabetes.

**Discussion:** Data analysis demonstrated that most adolescents reported being concerned about their ability to manage diabetes and felt motivated to check their BG and give insulin when hearing about the consequences of diabetes. We will continue to investigate this population, and we have decreased the A1c inclusion criteria to ≥8 to broaden our study group. This data will be used to help us improve our clinic visits in this population.

**Keywords:** adolescents, interventions, type 1 diabetes

## Pump it up: A clinic's journey to increasing insulin pump use

### Blake Adams, BSN; Grace Nelson, MD, Erica Davis, MSN, CDCES; Anna Heston, RN, CDCES; Kathryn Rogers, RN, CDCES

#### University of Tennessee Health Science Center, Le Bonheur Children's Hospital, Memphis, TN USA

Blake.Adams@lebonheur.org

**Background/Objective:** The Diabetes Clinic at Le Bonheur Children's hospital has made it a priority to focus on increasing use of insulin pumps. We were able to increase insulin pump use from 21% to 43% from Nov 2020 to July 2024.

**Methods:** We have worked to increase the use of insulin pumps with a multitude of changes within the clinic. We updated the PowerPoint slides presented in the mandatory pre pump education class. We also created an Insulin pump back up plan to be given to our patients, with the school care plan at the beginning of the school year. We revised the clinic's hypoglycemic protocol for patients on Hybrid Closed Loop pump, to accommodate for the algorithm and prevent rebound lows. Appointments are scheduled 2 weeks after pump start to troubleshoot any concerns and further educate when needed to help decrease adverse outcomes and overall adherence in new pump users.

**Results:** Baseline data showed that 21% of our patients were on an insulin pump as of November 2020 and as of July 2024, 43% of our patients are on an insulin pump. Our overall clinic insulin pump use has doubled in a three‐year timeframe.

**Conclusions:** Overall, we have seen improvement in our percentage of patients that are utilizing an insulin pump. Our goal is to increase pump usage to 50% by the end of 2024.

**Keywords:** diabetes education, diabetes technology, insulin pump

## Improvements in HbA1C since joining the exchange: One center's journey

### Grace Nelson MD, Blake Adams BSN

#### University of Tennessee Health Science Center, Le Bonheur Children's Hospital, Memphis, TN, USA

**Background/Objective:** Improving outcomes in People with T1D (PWD) can mean many different things. A metric often used is A1C. As we implement new methods of diabetes care we often rely on changes in A1C to confirm or validate our efforts. Le Bonheur Children's Hospital/University of Tennessee Joined the T1D Exchange QI consortium in 2020. Here we are pleased to share the changes in A1C averages across our entire patient population.

**Methods:** We examined HbA1Cs based on the lowest reported A1C in that year for patients diagnosed for more than 1 year and seen at least two times per year.

**Results:** In 2019 PWD and private insurance had A1C of 8.3, in 2023 this improved to 7.7%. In 2019 PWD and public insurance had A1C of 9.2, in 2023 this improved to 8.7.

In 2019 of PWD and public insurance, 53% age 0–4 had A1Cs between 7% to 10%, in 2023 this number increased to 67%. Those with A1C >10% went from 42% to 30%. Patients with public insurance age 13–17 went from 49% with A1C >10% to 40% between 2019 and 2023.

When looking at those with commercial insurance, PWD age 0–4 and A1C at target (<7%) from 15% to 33% between 2019 and 2023. Age 5–12 went from 17% to 28% at target.

**Conclusions:** Improvement in A1C is seen across all categories. During this time, we have increased CGM and insulin pump (Hybrid Closed loop) delivery.

## Improving pediatric diabetes care by unifying data analytics

### Alyssa Kramer, MLIS; Justin Indyk, MD, PhD; Don Buckingham, MBOE, CPHQ; Vijay Yeruva, MSE; Malak Abdel‐Hadi, MBOE, LSSBB; Manmohan Kamboj, MD

#### Alyssa Kramer, Nationwide Children's Hospital, Columbus, Ohio, USA

Alyssa.Kramer@nationwidechildrens.org

**Background/Objective:** Nationwide Children's Hospital (NCH) has significantly advanced data management by centralizing diverse data projects into a single data mart with consistent, well‐defined metrics and a user‐friendly dashboard. This unification has enhanced Diabetes data access, collection, and quality across clinical care, public dissemination, leadership oversight, and quality improvement (QI) initiatives.

Our Aim was to build on our institutional success in data unification by collaboration with data analytics experts to create seamless and accurate data resources, including (1) an Endocrinology/Diabetes‐specific data mart and (2) an interactive data dashboard.

**Methods**: NCH has gradually been transitioning to a unified data approach, prioritizing shared data sets and architectures over siloed data sources. This shift supports quality improvement goals across pediatric chronic illness conditions, enhancing usability and cross‐program applicability for making evidenced based interventions in diabetes patient care.

**Results**: Ongoing outcomes include reduced data collection downtime, faster report turnaround times, enhanced quality access, the ability to cross‐verify internal and external data, and support for a broader range of projects with increased consistency and clarity in data definitions. A notable example was our development of a Type 1 Diabetes composite score* measuring patient care outcomes, illustrating our commitment to uniform data usage.

**Conclusions**: Our journey from scattered data and definitions to a unified data source marks a significant transformation. We continue to refine our metrics and methods, aiming for comprehensive data coherence and utility across our institution, furthering our commitment to improving pediatric diabetes care.

**Keywords:** data science; endocrinology; diabetes; quality improvement

*The Type 1 Diabetes Composite Score: An Innovative Metric for Measuring Patient Care Outcomes Beyond Hemoglobin A1c. (*Pediatric Quality & Safety*, DOI: 10.1097/pq9.0000000000000354)

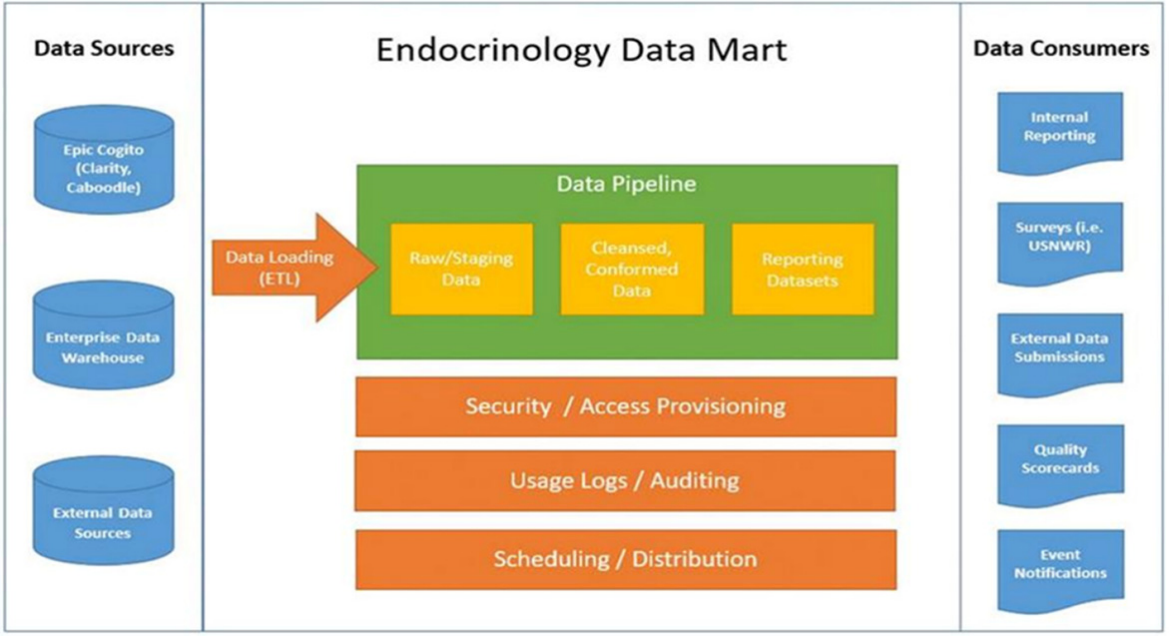

## Pneumococcal polysaccharide vaccination in patients with type 1 diabetes mellitus

### Jacob N. Miller, DO; Naomi Fogel, MD; Monica E. Bianco, MD

#### McGaw Medical Center of Northwestern University, Ann & Robert H. Lurie Children's Hospital of Chicago, Chicago, IL, USA

jamiller@luriechildrens.org

**Background/Objectives:** The CDC recommends vaccination for patients with type 1 diabetes mellitus (T1D) with PPSV23, an additional pneumococcal vaccination not included in the standard vaccine schedule, due to their increased risk for invasive pneumococcal disease. We suspect many patients with T1D are not receiving this recommended vaccine.

**Methods:** We queried the Lurie Children's Hospital T1D Registry of all active T1D patients for vaccination status with the PPSV23 vaccine. In June 2024, the practice implemented a PPSV23 vaccine section in the diabetes note template. If vaccinated anywhere, the date is automatically populated. If not, a message prompts the provider stating: “This vaccine is due and should be arranged through the pediatrician's office.”

**Results:** There were 1237 active patients in the T1D Registry in July 2024. Of these, 36 had documented vaccination with the PPSV23 vaccine at baseline. Since the addition of the PPSV23 vaccine status to the note template in June 2024, no additional patients were vaccinated.

**Conclusions:** The PPSV23 vaccination rate in our clinic is very low. Since integrating vaccine reminders into the clinical workflow, no additional vaccines were given, but it may be too soon to assess the impact of this intervention since it requires families to return to their pediatrician's office for vaccine administration. Future PDSA cycles may include a provider prompt in the note, inclusion in the after‐visit summary, or partnering with the hospital to provide the vaccine on site.

**Keywords:** immunization; vaccination; 23‐valent pneumococcal capsular polysaccharide vaccine; diabetes mellitus, type 1; pediatrics.

## Analysis of T1DM patient additional training experience on omnipod 5 pump: Survey‐based

### Kaveri Bhargava, BA; Marina Basina, MD

#### Stanford University School of Medicine, Stanford, CA, United States of America

kaverib@stanford.edu

**Background/Objective:** Given the recent FDA‐approval of Omnipod 5 pump system (OP5), there is little information regarding its best pump training practices. A study presented by Berget et al. at the 2024 American Diabetes Association Scientific Sessions evaluating glycemic outcomes based on training type. Our study surveyed patients to evaluate their educational needs, training satisfaction, and need for additional pump training sessions.

**Methods:** Following IRB approval, a REDCap survey was emailed to patients of an outpatient Endocrinology clinic. Patients over 18 years with Type 1 Diabetes on OP5 were included. Patients who opted for additional training sessions were offered three group sessions, two virtual and one in person.

**Results:** Out of 181 invitations sent, 41 survey responses were received (23.0% response rate). The formally trained (FT) (27/41; 65.9%) and self‐trained (ST) (14/41; 34.1%) groups expressed similar patient satisfaction. Of the 18/41 patients who requested additional training, 6 patients completed sessions. Average score on post‐test assessment of patient knowledge from training was 93.8%.

**Conclusions:** Our study demonstrated a) similar patients' satisfaction with ST and FT methods. b) Patients benefitted from optional additional training. Patient satisfaction with ST has not been previously evaluated. Considering individual variability in learning needs, different assimilation of new information, availability of online materials, allocation of the resources, and time constraints, ST can be a viable option for some patients with additional follow up or ongoing training sessions as needed. Larger and longer studies are needed.

**Key Words:** Omnipod 5, Patient Education, Type I DM

## Nurse driven diabetic ketoacidosis fluid titration protocol

### Michelle Jeski MSN, RN, PCNS‐BC, Emilie Hess MS, Margaret Anderson B Pharm, BCPPS, Alana Guidetti BSN, RN, David Hansen MD, MPH, Abeir Mohamed MD, Melissa Schafer MD, Angela Wratney MD, Roberto Izquierdo MD

#### 1SUNY Upstate Medical University, Syracuse NY, USA

**Objective:** Decrease the time from blood glucose to rate adjustment from 16 to <5 min within 3 months in pediatric patients in Diabetes Ketoacidosis (DKA) admitted to the hospital.

**Methods:** A RN driven protocol was developed to allow nurses to titrate the rates of the 2‐bag system. The focus population was patients with DKA, aged 2–18 years on 1.5x maintenance fluids outside of the ICU. Children with a mixed picture concerns for cerebral edema or requiring critical care were excluded. Using our existing DKA policy and guidelines, a calculator was built into the EMR which identifies the rate change needed based on the entered blood glucose. Time to rate change was collected through EMR report, and complications assessed through a unit‐based tracking system.

**Results:** Twelve patients have been treated using the protocol with an average time to rate change of 5 minutes. Time on insulin has remained unchanged at 14.4 h. No complications have been reported.

**Conclusions:** This nurse driven protocol has been effective in streamlining care for our patients with DKA outside of the ICU. Nurses and residents stated increased satisfaction with the RN driven protocol as care is more efficient. Future steps include expanding the protocol to other units and patients with DKA on alternative fluid regimens, as well as assessing other factors that influence time on insulin.

**Keywords**: pediatrics, diabetic ketoacidosis, type 1 diabetes

## Insulin pump initiation: effects at children's of Alabama

### Marinés Castillo Echevarría, MD^1^ , Jessica Schmitt, MD, MSHQS^1^

#### ^1^University of Alabama at Birmingham, Birmingham, AL, USA, 35233

mcastilloechevarria@uabmc.edu

**Background:** We aimed to better understand the effects of our current system by evaluating insulin pump initiation after attending “Pre‐pump class” at Children's of Alabama. Class attendance is required prior to coverage of an insulin pump for patients insured by Alabama Medicaid but is optional for those with non‐Medicaid insurance.

**Methods:** A retrospective review of attendees to pre‐pump class from January 2022 to July 2023 was completed. Patients who initiated an insulin pump training session prior to January 2024 were identified as “pump starters (PS)”. Patients who did not were identified as “non‐starters (NS).” Demographic and medical data were compared.

**Results:** A total of 283 patients aged 1–19 attended pre‐pump class during the period of study. Of these, 187 patients (66%) started an insulin pump. PS and NS differed in race, age, insurance, duration of diabetes, and pre‐pump hemoglobin A1c (see Table 1). PS preferred untethered systems, with 131 (70.0%) selecting an untethered pump. Most PS used a pump as an automatic insulin delivery (AID) system (*n* = 116, 62% of PS). Insurance and race were *not* associated with selecting an AID (*p* = 0.13 and 0.65). Median duration from pre‐pump attendance to pump initiation was 108 days (interquartile range 76–154).

**Conclusions:** While race and insurance are not associated with selection of an AID system compared to traditional insulin pump system, they are associated with PS versus NS after attending pre‐pump class. Future work will aim to reduce these disparities and increase access for all interested in diabetes technology.

**Key Works:** Child; Diabetes Mellitus, Type 1; Healthcare disparities; Insulin infusion systems

**Table:** Table 1. Characteristics of pre‐pump attendees and comparison of those who did and did not initiate an insulin pump.

	Total *n* = 283	Pump starters *n* = 187	Non‐starters *n* = 96	Comparison
Sex: Female (%)	148 (52.3)	99 (52.9)	49 (51.0)	0.8
Race:				0.039
NHW	208 (73.5)	147 (78.6)	61 (63.5)	
NHB	58 (20.5)	32 (17.1)	26 (27.1)	
Hispanic	9 (3.2)	5 (2.7)	4 (4.2)	
Other	8 (2.8)	3 (1.6)	5 (5.2)	
Language: Non‐English (%)	6 (2.1)	3 (1.6)	3 (3.1)	0.4
Insurance: Private (%)	138 (48.8)	108 (57.8)	30 (31.3)	<0.001
Pre‐pump Age	11.4 (4.1)	11.0 (4.0)	12.2 (4.1)	0.015
Age at DM Dx	8.8 (4.1) (*n* = 280)	8.7 (4.0) (*n* = 185)	9.0 (4.2) (n = 95)	0.6
Duration of DM at pre‐pump	2.5 (3.1)	2.2 (3.1)	3.2 (3.1)	<0.001
Hemoglobin A1c prior to pre‐pump attendance	8.1 (7.1–9.6)	8.0 (6.9–9.1)	8.6 (7.5–10.4)	0.002

Data are expressed as mean (SD), n (%), and median (interquartile range). NHW: non‐Hispanic white; NHB: non‐Hispanic Black; DM: diabetes mellitus.

## Barriers to the use of technology in type 1 diabetes

### Maria Adriana Yanez Bello, MD; Maria Borja Pesantez, MD; Maddison Saalinger, RD CDCES; Alex Macias, BS; Simran Shikh, APRN; Michael Garcia, MD; Tatiana Nisenboym, BS; Aleida Saenz, APRN CDCES; Francesco Vendrame, MD PhD

#### Division of Endocrinology, Diabetes, and Metabolism, University of Miami, Miami, FL, USA

mariayanezb09@gmail.com

**Background/objective:** In type 1 diabetes (T1D) the use of automated insulin delivery systems (AID) is associated with reduced HbA1c, improved time in range and reduced risk of hypoglycemia. Despite such benefits some people remain on multiple daily insulin injections (MDI) with or without the use of a continuous glucose monitoring system (CGM). The objective of the present study was to assess the use of AID and the barriers to its use in our clinic.

**Methods:** We administered a questionnaire to 196 established T1D patients capturing information about demographics, diabetes management and answers to 19 questions addressing barriers to the use of technology. Data are expressed as mean ± SD. A two‐sided *p* = 0.05 was considered statistically significant.

**Results:** Patients' age was 40.3 ± 14.9 years, 73.7% were Hispanic and 26.3% non‐Hispanic White, 89% had diabetes duration >5 years. HbA1c was 7.1 ± 0.9% in the AID group and 7.8 ± 1.6% in the group on MDI + CGM (*p* = 0.0020). This group presented more barriers in the use of technology compared to people with AID (*p* < 0.0001) with the top 5 barriers represented by 1) cost of supplies, 2) nervous to rely on technology, 3) cost of device, 4) insurance coverage, 5) wearing devices all the time.

**Conclusions:** In a largely Hispanic population with T1D the use of MDI + CGM was associated with worse glycemic control and a higher number of barriers in the use of technology compared to people with AID. Strategies to address modifiable barriers such as the reliance on technology are needed.

**Key words:** type 1 diabetes, automated insulin delivery systems, barriers.

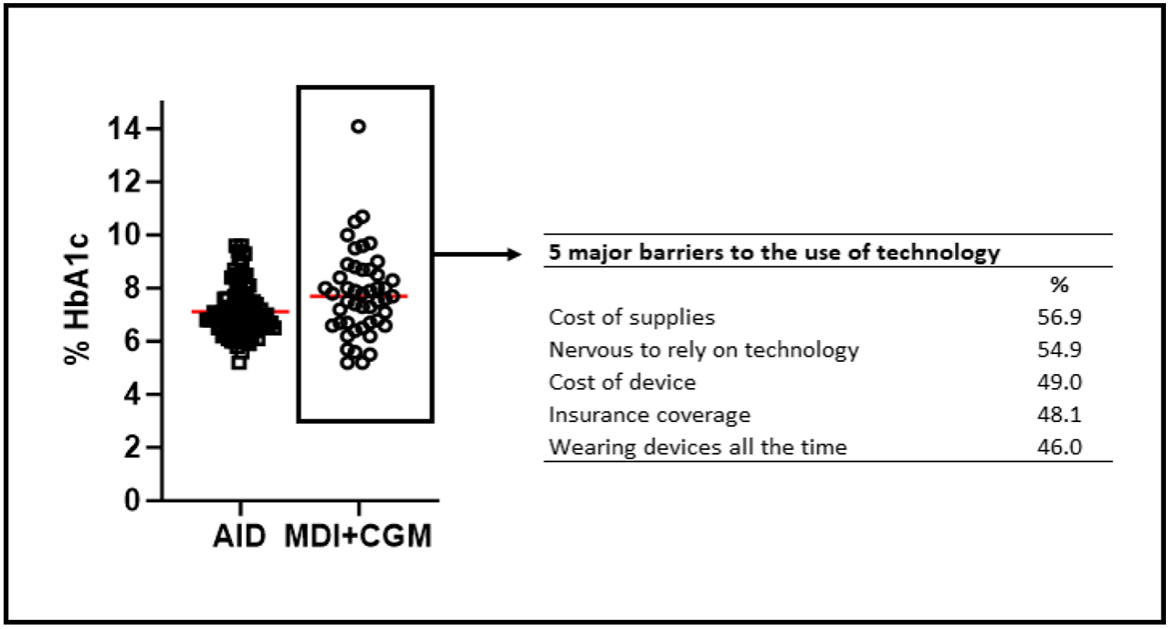

## Fostering connectivity by increasing data sharing to facilitate remote patient monitoring

### Abha Choudhary, MD; Marsha Mackenzie, RD; Katherine Hamilton, RN

#### Children's Medical Center, University of Texas Southwestern, Dallas, Texas, USA

abha.choudhary@utsouthwestern.edu

**Background:** Technology is reshaping the way physicians and Certified Diabetes Care and Education Specialists view glucose values, measure diabetes outcomes, and collaborate with their patients to obtain optimal outcomes. Data review from December 2023–February 2024 showed that only 60% of our patients utilizing Dexcom continuous glucose monitor (CGM) in the Dallas campus were sharing data to Clarity. We developed a quality improvement initiative to increase rates of data sharing to facilitate remote patient monitoring.

**Methods:** The aim is to increase CGM data sharing between type 1 diabetes families and clinician staff by 15% over a 6‐month timeframe compared to baseline. We utilized quality improvement tools including fish bone, effort impact matrix, key driver diagram to look at barrier to CGM sharing. We developed PDSA cycles which utilized the medical assistants and clinic nurse to facilitate sharing using smart phrases and patient instructions.

**Results:** We started PDSA cycle in April 2024. We noted many barriers to CGM sharing. See p chart for the CGM utilization.

**Conclusions:** The patients who utilize receivers due to no phones or incompatible phones and patients who were on automated insulin pumps were not sharing to Clarity. We will develop additional PDSA cycles to overcome these barriers.

**Keywords:** continuous glucose monitoring, sharing, remote patient monitoring

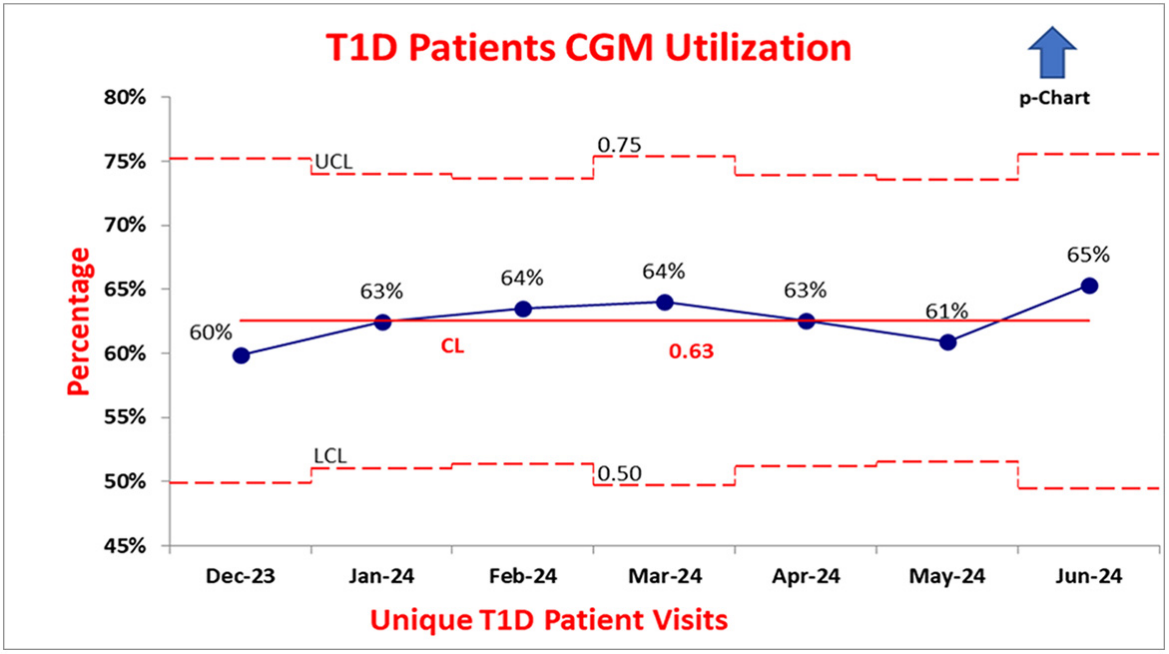

## Increasing scheduling of adult diabetes care prior to graduation from pediatric diabetes care for patients with type 1 diabetes

### Ryan Canter, MD^1^ ; Vandana Raman, MD^1^ ; Stephanie Sund, MSN, RN; Janet Sirstins, RN, BSN, CDCES; Aileen Edwards, RN, BSN, CDCES; Megan Counter, MSW, CSW; Billie Whitaker, CMA III; Tina Wadhwa, MS; Marie Couldwell, MD; Allison Smego, MD^1^

#### ^1^Division of Pediatric Endocrinology, University of Utah, Salt Lake City, UT, USA

ryan.canter@hsc.utah.edu

**Background:** Delay in establishing adult diabetes care is common among emerging adults (EAs) with type 1 diabetes (T1D), and is associated with higher HgA1c, increased emergency visits, and higher rates of hospitalization for diabetic ketoacidosis. We aim to increase the percentage of patients who have a scheduled appointment with adult diabetes care prior to graduation from our pediatric diabetes center.

**Methods:** This quality improvement (QI) project was conducted in a large, pediatric diabetes center by a multidisciplinary team. QI methodology informed the design and iterative testing of interventions.

**Results:** Prior to this initiative, healthcare transition appointments were not documented in our center. After implementation of a new tracking system, it became apparent that many patients were leaving our center without scheduled adult care. With targeted nurse care coordination, the percentage of patients with a documented scheduled adult diabetes appointment has ranged from 16% to 42% by month. With a change in our transition policy that prompts return to our pediatric clinic if no adult appointment is scheduled, the percentage of patients rescheduling in our center increased from 16% to 54% during that same time.

**Conclusion:** Standardizing the tracking of adult diabetes care appointments helps identify EAs who are at risk of interrupted diabetes care. For EAs without scheduled adult care, returning to the pediatric setting and utilizing targeted nurse care coordination prevents interrupted care and improves scheduling of adult diabetes care.

**Keywords:** transition to adult care; diabetes mellitus, type 1; healthcare transition; quality improvement; patient care team

## Increasing automated insulin delivery system use in pediatric patients with type 1 diabetes

### Kimberly Vidmar, MD; Whitney Beaton, MSN, RN, ACCNS‐P, CDCES; Rachel Fenske, PhD, RDN, CD, LDN, CDCES; Tracy Bekx, MD; Juliana Price, MSN, RN, CDCES; Elizabeth Mann, MD

#### University of Wisconsin School of Medicine and Public Health, Department of Pediatrics, Division of Endocrinology and Diabetes, Madison, Wisconsin, USA

kvidmar@wisc.edu

**Background/Objective:** Automated insulin delivery systems improve glycemic outcomes and quality of life for people with type 1 diabetes (T1D). Our objective is to increase the percentage of pediatric patients with T1D using insulin pumps from 55% to 65% by May 2025, while reducing disparities in pump use by race/ethnicity and insurance type.

**Methods:** Barriers to pump use were assessed by surveying patients not using insulin pumps and diabetes team staff. Barriers identified, including access to initial pump education and standardized pump start criteria, informed key driver diagram development. Initial Plan‐Do‐Study‐Act (PDSA) cycles included an introduction of a diabetes technology fair.

**Results:** Since completion of two monthly technology fairs, 18 individuals and their families attended; 13 (72%) ordered insulin pumps. All technology fair attendees were non‐Hispanic white, most privately insured, with an average time since diagnosis of 2 years, and a mean A1c of 8.94%. Families reported high levels of satisfaction with the technology fair content and format. The percentage of patients using insulin pumps, assessed by p‐chart, is increasing but not statistically significant (Figure 1).

**Conclusions:** Providing initial insulin pump education in a group setting via a technology fair is acceptable to patients and families and opens up diabetes educator clinic availability but does not address disparities. Future PDSA cycles will standardize distinct pump start pathways to offer individualized support and better address disparities. Other aspects of this work include stakeholder education, pump introduction at T1D diagnosis, and improving workflow for pump ordering process.

**Keywords:** type 1 diabetes, pediatric, diabetes technology, social disparities in health, insulin pump

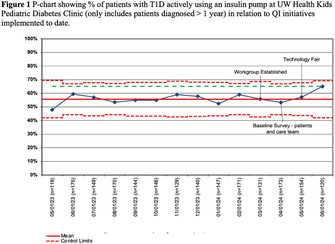

## Diabetes pediatric annual visit

### Isabel Reckson, RD, CDCES, MPH; Alyson Weiner, MD; Elizabeth Gunckle, MSN, RN, CPNP‐PC; Emily Coppedge, MSN, RN, CPNP‐PC, CDCES; Zoltan Antal, MD

#### Weill Cornell Medicine, New York, NY, USA

isr2007@med.cornell.edu

**Background/Objective:** The American Diabetes Association (ADA) recommends routine social work and nutrition care for all patients with type 1 diabetes (T1D) for diabetes management success. Our team hypothesized that implementing a comprehensive annual visit would allow our patients the opportunity to see interdisciplinary providers as well as be screened for important topics such as depression, disordered eating, diabetes distress, and social determinants of health (SDOH).

**Methods:** The annual visit framework includes appointments with the diabetes provider, the dietitian/Certified Diabetes Care and Education Specialist (CDCES), and the social worker. The visit includes annual ADA labs and multiple screeners to better understand mental health and barriers to T1D care. Depression is evaluated using the Patient Health Questionnaire (PHQ‐9), diabetes distress using the Problem Areas in Diabetes Teen and Child (PAID‐T and PAID‐C) questionnaires, and disordered eating using the Diabetes Eating Problems Survey Revised (DEPS‐R). The SDOH screener was adapted from Le Bonheur's Children's Hospital.

**Results:** Since February 2024, we have completed 15 annual visits, which represents 9% of our patient population. Ten of the 15 visits had some components which were not completed due to several factors, including availability of our social worker and distribution of necessary screeners.

**Conclusions:** The annual visit provides an opportunity for comprehensive diabetes care, addressing barriers to diabetes management and recognizing needs that may not otherwise be identified. Future efforts to increase the number of annual visits will include streamlining staff schedules and simplifying the screening distribution process.

**Keywords:** diabetes mellitus type 1, pediatrics

